# Rilmenidine extends lifespan and healthspan in *Caenorhabditis elegans* via a nischarin I1‐imidazoline receptor

**DOI:** 10.1111/acel.13774

**Published:** 2023-01-20

**Authors:** Dominic F. Bennett, Anita Goyala, Cyril Statzer, Charles W. Beckett, Alexander Tyshkovskiy, Vadim N. Gladyshev, Collin Y. Ewald, João Pedro de Magalhães

**Affiliations:** ^1^ Integrative Genomics of Ageing Group Institute of Ageing and Chronic Disease, University of Liverpool Liverpool UK; ^2^ Department of Health Sciences and Technology, Laboratory of Extracellular Matrix Regeneration Institute of Translational Medicine, ETH Zürich Schwerzenbach Switzerland; ^3^ Division of Genetics, Department of Medicine Brigham and Women's Hospital, Harvard Medical School Boston Massachusetts USA; ^4^ Belozersky Institute of Physico‐Chemical Biology Moscow State University Moscow Russia; ^5^ Present address: Institute of Inflammation and Ageing University of Birmingham, Queen Elizabeth Hospital Birmingham UK

**Keywords:** aging, autophagy, drug repurposing, longevity, mTOR, nischarin receptor

## Abstract

Repurposing drugs capable of extending lifespan and health span has a huge untapped potential in translational geroscience. Here, we searched for known compounds that elicit a similar gene expression signature to caloric restriction and identified rilmenidine, an I1‐imidazoline receptor agonist and prescription medication for the treatment of hypertension. We then show that treating *Caenorhabditis elegans* with rilmenidine at young and older ages increases lifespan. We also demonstrate that the stress‐resilience, health span, and lifespan benefits of rilmenidine treatment in *C. elegans* are mediated by the I1‐imidazoline receptor *nish‐1*, implicating this receptor as a potential longevity target. Consistent with the shared caloric‐restriction‐mimicking gene signature, supplementing rilmenidine to calorically restricted *C. elegans*, genetic reduction of TORC1 function, or rapamycin treatment did not further increase lifespan. The rilmenidine‐induced longevity required the transcription factors FOXO/DAF‐16 and NRF1,2,3/SKN‐1. Furthermore, we find that autophagy, but not AMPK signaling, was needed for rilmenidine‐induced longevity. Moreover, transcriptional changes similar to caloric restriction were observed in liver and kidney tissues in mice treated with rilmenidine. Together, these results reveal a geroprotective and potential caloric restriction mimetic effect by rilmenidine that warrant fresh lines of inquiry into this compound.

## Graphical abstract

Rilmenidine has prolongevity and health preserving effects in *C. elegans*, increased stress resistance and increased autophagy. The stress‐resilience, healthspan, and lifespan benefits of rilmenidine treatment in *C. elegans* are mediated by the I1‐imidazoline receptor nish‐1.

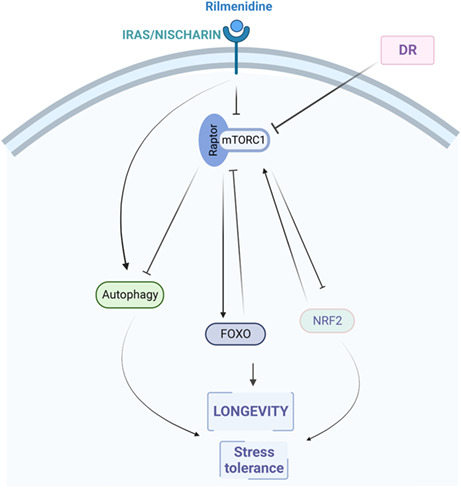

## 1 INTRODUCTION

Individuals over 65 are now the fastest‐growing demographic group worldwide, a fact that emblematizes the global aging population (Jarzebski et al., 2021). Unfortunately, at present, with age comes age‐related chronic disease and death (Fontana et al., 2014), and as such, the estimated benefits of delaying aging, even if the effect is rather small, are immense (de Magalhães et al., 2012; Farrelly, 2010; Goldman et al., 2013). A large body of evidence has demonstrated that the aging rate can be markedly slowed in model organisms. So far, caloric restriction (CR) is the most robust antiaging intervention (Liang et al., 2018), and CR promotes longevity across species (Fontana et al., 2014). However, studies of CR in humans have had mixed results, low compliance, and many side effects (Most et al., 2017), meaning finding medications that can mimic the effect of caloric restriction is the most reasonable antiaging target (Ingram & Roth, 2015). However, only a few compounds have been identified to mimic the beneficial effects of CR.

Previously, we detailed a drug repositioning method in which we identified potential caloric restriction mimetics (CRMs), yielding biologically relevant results (Calvert et al., 2016). We did this by comparing drug‐gene signatures to the in vitro transcriptome of caloric restriction, looking for drugs with an overlapping profile. One drug identified during this work was allantoin, which was further confirmed to extend lifespan in *Caenorhabditis elegans* (Admasu et al., 2018). Allantoin, however, does not display oral bioavailability, obviating its use in humans (Kahn & Nolan, 2000). Moreover, we propose that the prolongevity effect of allantoin could be mediated by an affinity to the imidazoline type 1 receptor (I1R) expressed on a yet to be discovered nischarin ortholog in *C. elegans*. Imidazoline receptors do not show affinity toward catecholamines, but instead bind imidazoline and guanidinium compounds of which allantoin is a derivative of the latter (Dardonville & Rozas, 2004). Therefore, a more potent and specific agonist of the imidazoline receptor, such as the widely prescribed, oral antihypertensive rilmenidine could have a similar, if not better, longevity effect in *C. elegans* but with more potential for future translatability to humans. This is supported by our previous work showing rilmenidine to elicit a similar transcriptional profile to CR (Calvert et al., 2016), to reprogram human cell transcription profiles to a more youthful state (Statzer et al., 2021) and to induce a gene expression signature in the liver of mice similar to CR (Tyshkovskiy et al., 2019).

Herein, we show rilmenidine administration extended lifespan in *C. elegans* when commenced from early adulthood or only once the animal had aged. Furthermore, it decelerated the development of decrepitude, without altering developmental periods. Lifespan extension was not possible in genetic models of CR (*eat‐2*), suggesting a longevity effect was potentially induced through CR mimicry. Moreover, this was supported by rilmenidine‐induced lifespan effects being dependent on key CR nexuses: DAF‐16, TOR, and NRF1,2,3/SKN‐1 (Blackwell et al., 2015, 2019). In *Caenorhabditis elegans*, rilmenidine elicited increases in ERK activity, typical of in vitro imidazoline agonist exposure, which was abrogated following blockade of an imidazoline‐binding site. This effect was mimicked following the knockout of *f13e9.1*, which was characterized herein as the nematode ortholog of the human imidazoline type 1 receptor (IRAS), and renamed *nish‐1*. Indeed, rilmenidine also increased nematode thermotolerance as well as autophagy, both dependent on imidazoline binding, and demonstrated a capacity to attenuate the accumulation of polyQ aggregates. Lastly, we find that treating mice with rilmenidine showed transcriptional changes in liver and kidney similar to caloric restriction. Overall, our findings reveal rilmenidine as a potential caloric restriction mimetic and as a novel geroprotective compound.

## 2 RESULTS

### 2.1 Rilmenidine improves survival in *Caenorhabditis elegans*

**FIGURE 1:**
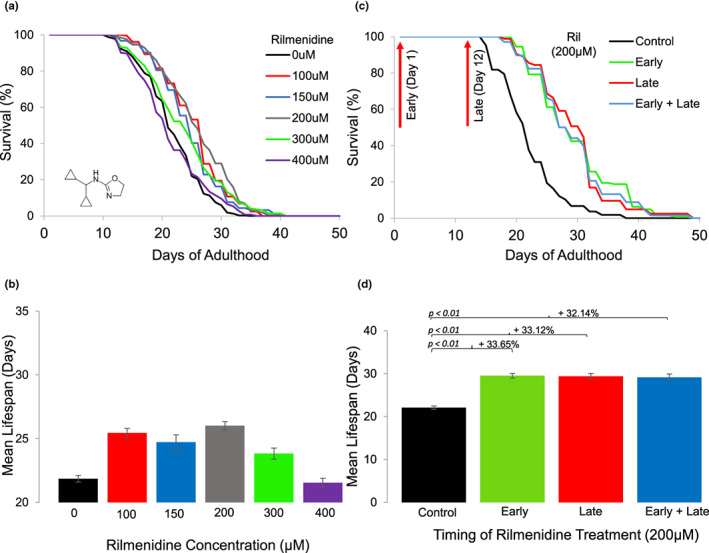
Improved survival of *C. elegans* treated with rilmenidine. (a) Pooled survival curve showing significant lifespan extension in WT animals treated with rilmenidine at all concentrations, except 400 μM, compared to 1% DMSO vehicle control. (b) Bar graph showing quantified lifespan data in terms of mean lifespan (days) for each rilmenidine concentration (100–400 μM) treated WT, compared to DMSO vehicle control; 200 μM concentration of rilmenidine provides maximum lifespan increase (19%) in WT. (c) Pooled survival curve showing late‐life treatment with rilmenidine (200 μM) at day 12 of adulthood increased lifespan in WT compared to DMSO vehicle control. (d) Quantified data for lifespan assay showing percentage increase in mean lifespan of adult WT‐fed 200 μM rilmenidine at different times (day 1 or day 12 adulthood) compared to DMSO vehicle control. Error bars represent SEM; adjusted *p*‐value was derived from log‐rank test and Bonferroni correction. Kaplan–Meir survival analysis was performed on pooled data from at least three independent trials. Quantitative data and statistical analyses for the representative experiments are included in Table S1.

Previous computational studies predicted rilmenidine as a longevity drug and CR mimetic (Calvert et al., 2016; Tyshkovskiy et al., 2019). To test if rilmenidine is involved in lifespan regulation, we treated wild‐type (WT) *Caenorhabditis elegans* with a range of concentrations (0, 100, 150, 200, 300, and 400 μM) of rilmenidine from larval stage L4 and performed lifespan assays with UV‐killed OP50 *E. coli* until dosage no longer elicited a significant response (Sutphin & Kaeberlein, 2009). We found that rilmenidine extended the lifespan of WT animals by approximately 19% compared to DMSO‐treated WT control, at an optimal concentration of 200 μM (Figure 1a,b, Table S1). Lifespan extension was no longer observed in *C. elegans* treated with 400 μM rilmenidine. In addition, moxonidine belonging to the class of imidazoline agonists was also predicted as a caloric restriction mimetic (Calvert et al., 2016); however, moxonidine could only slightly (7.1%) increase lifespan of WT *C. elegans* (Figure S1, Table S1). To ensure prolongevity effects were not mediated through an induction of calorie restriction and altered feeding behavior, we monitored feeding and pharyngeal pumping in Day 3 animals, treated with 200 μM rilmenidine at L4 larval stage. Drug treatment did not affect consumption of RFP beads nor reduce pharyngeal pumping compared to control‐treated animals (Figure S1).

Rilmenidine should ideally work when administered later in life. Researchers have been keen to establish if potential compounds can extend lifespan when treatment is initiated later in life and the aging process has considerably progressed (Cabreiro et al., 2013; Guha et al., 2014). This is particularly important given the difficulties of eliciting CR‐mediated longevity in aged animals. Both mice and rats initiated on CR in late life often results in negligible effects or indeed reductions in longevity (Forster et al., 2003; Lipman et al., 1998, 1995; Ross, 2009). Furthermore, current CRMs such as metformin, when administered at day 10 of *C. elegans'* adulthood or 20 months in mice, instead shortened lifespan and accelerated age‐related pathologies (Espada et al., 2020; Zhu et al., 2021). Interestingly, we found that in *C. elegans*, rilmenidine prolonged the lifespan to the same extent (approximately 33%) whether exposure to the drug was initiated during youth, day 1 of adulthood, or when old, day 12 of adulthood (Figure 1c,d, Table S1). However, this prolongevity effect was not additive to lifespan extension exerted by early life treatment, suggesting a possible cellular mechanism that upon activation lasts through adulthood and aging (Figure 1c,d, Table S1). These results indicate that rilmenidine might be employed as a pharmacological intervention during old ages to extend lifespan.

### 2.2 Rilmenidine acts as a potential CR mimetic

**FIGURE 2:**
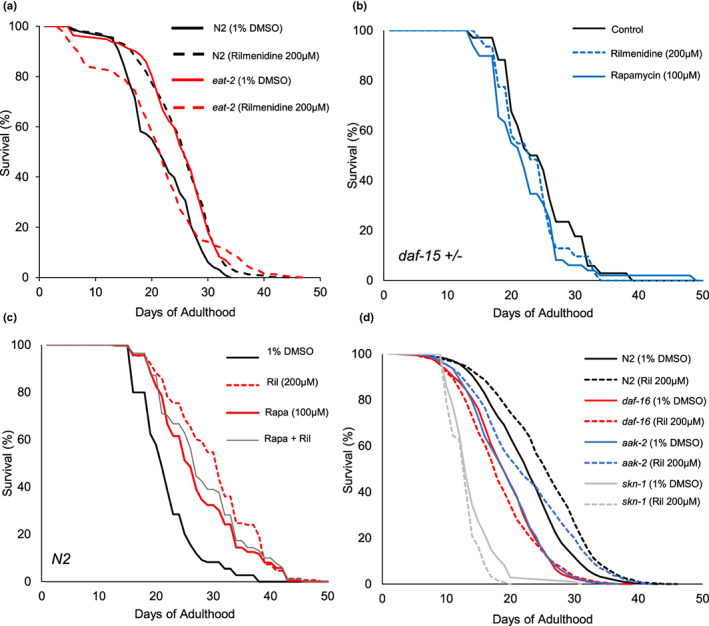
Effects of rilmenidine treatment on survival of CR‐associated mutants. Survival curves showing the inability of rilmenidine to extend life span in (a) DR1116 *eat‐2(ad1116)* mutants, (b) DR412 *daf‐15(m81/+)* mutants, (c) rapamycin‐treated WT and (d) GR1307 *daf‐16(mgDf50)*, and LD1057 *skn‐1(tm3411)*, but not in TG38 *aak‐2(gt33)* mutants. Raw data, quantitative data, additional trials, and statistical analyses for the representative experiments are included in Table S1.

Rilmenidine shares the transcriptome profile with CR, as indicated by our meta‐analysis studies (Calvert et al., 2016; Tyshkovskiy et al., 2019). Therefore, to discern the signaling pathway through which rilmenidine extends lifespan, we chose a genetic (*eat‐2* mutant) model of CR in *C. elegans* (Lakowski & Hekimi, 1998). Rilmenidine did not further increase the longevity of the *eat‐2* mutants (Figure 2a, Table S1). CR is thought to work through the nutrient sensor, mTOR complex 1, mTORC1 to extend lifespan (Hansen et al., 2007; Statzer et al., 2022). To investigate the requirement of mTOR in lifespan increase by rilmenidine, we performed lifespan assays using a heterozygous mutant of *daf‐15*, the raptor adaptor protein in mTORC1. We found that rilmenidine could not increase the longevity additively of the *daf‐15(m81/+)* mutants (Figures 2b, S2, Table S1). Complementary, we inhibited mTOR pharmacologically by rapamycin. Rapamycin significantly increased the lifespan of WT animals. Co‐treatment with rilmenidine and rapamycin failed to further increase the mean lifespan extension of WT animals treated either with rilmenidine or rapamycin individually (Figures 2c, S2, Table S1). This result strengthens the earlier observation that rilmenidine may act through mTOR1 signaling for increasing the lifespan.

In *Caenorhabditis elegans*, mTOR inhibition by genetic or pharmacologic intervention, like in mammals, leads to activation of key longevity transcription factors such as SKN‐1/NRF2 and DAF‐16/FOXO (Robida‐Stubbs et al., 2012). Moreover, on solid media, *daf‐16* is indispensable for CR‐induced longevity (Greer et al., 2007). To explore whether rilmenidine‐induced longevity effects are DAF‐16 and SKN‐1 driven, we employed *skn‐1(tm3411)* and *daf‐16(mgDf50)* null mutants to perform lifespan assays. We found that rilmenidine did not significantly extend the lifespan of either *daf‐16* or *skn‐1* mutants, revealing a requirement of *daf‐16* and *skn‐1* for longevity benefits by rilmenidine (Figure 2d, Table S1).

AMPK is another critical metabolic switch, which works antagonistically with the mTOR pathway and is activated by CRM drugs like metformin (Onken & Driscoll, 2010a, 2010b; Zhang et al., 2019). Thus, we assessed the necessity of AMPK in the mediation of rilmenidine's prolongevity effects. We found that rilmenidine additively extended lifespan in *aak‐2 (gt33)* mutants compared to WT, suggesting AMPK is not required for lifespan regulation by rilmenidine (Figure 2d, Table S1). Taken together, it can be concluded that rilmenidine's life‐extension effects are not additive to genetic dietary restriction and the key CR genes, ceTOR, *daf‐16*, and *skn‐1* function in the prolongevity of rilmenidine.

### 2.3 Autophagy is required for extension in lifespan by rilmenidine

CR is routinely cited as the most robust physiologic inducer of autophagy and nutrient depletion is the gold standard for autophagic induction in culture (Kroemer et al., 2010). Thus, autophagy is a vital component of CR‐induced longevity. Multiple models have demonstrated that the longevity benefits of dietary restriction are abrogated following the knockdown of autophagic machinery (Hansen et al., 2007; Jia & Levine, 2007; Rubinsztein et al., 2011). As such, researchers often seek to demonstrate that their CRM of interest upregulates autophagy and that this upregulation mediates its lifespan extension (Eisenberg et al., 2009; Pietrocola et al., 2018; Shintani et al., 2018).

**FIGURE 3:**
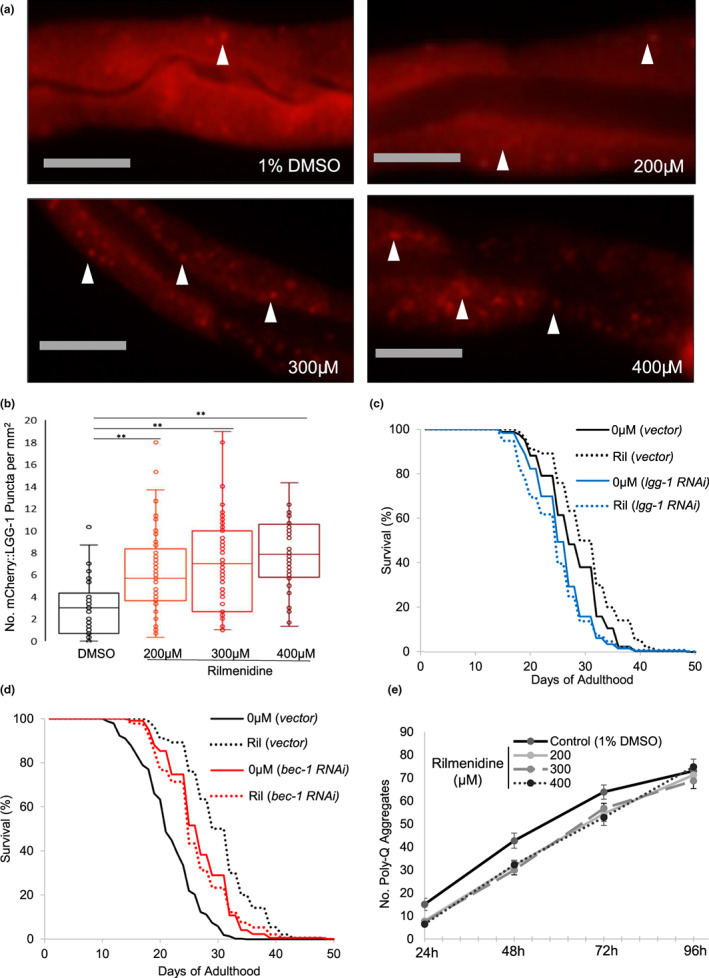
Induced autophagy by rilmenidine perturbed polyQ aggregation. (a) Representative images of day 2 adult transgenic animals, expressing the intestinal specific autophagy reporter gene P*nhx‐2::*mCherry::lgg‐1 showing increased autophagy, when exposed to varying concentrations of rilmenidine for 24 h compared to 1% DMSO vehicle. Arrows indicate autophagosome puncta formation. Scale bar = 20 μm. (b) The graph shows the interquartile distribution of the mean number of mCherry::LGG‐1 puncta in the posterior intestine of the animals in each condition. Error bars, upper: Q3 + 1.5*IQR; minimum: Q1–1.5*IQR. **p < 0.01, *p < 0.05; one‐way ANOVA followed by a Tukey's post hoc test. (c, d) Inhibition of autophagy abrogates the prolongevity effect of rilmenidine as shown by survival curves of WT animals fed either RNAi bacteria expressing an empty vector (L4440), or *lgg‐1* (c) or *bec‐1* (d) dsRNA from day 1 adulthood in the presence or absence of 200 μM rilmenidine. Kaplan–Meir survival analysis was performed on pooled data from at least three independent trials. Groups tested by log‐rank with Bonferroni correction; *p* < 0.05. Quantitative data and statistical analyses for the representative experiments are included in Table S1. (e) The graph depicts quantified data of Q40::YFP aggregates in body wall muscles per entire animal after treatment with differing rilmenidine concentrations for the indicated times from L1. Data represented as the pooled mean number of aggregates per animal. Error bars are ± SEM. Significance derived from two‐way repeated‐measures ANOVA *p* < 0.05.

Rilmenidine has been shown to induce autophagy in SOD1‐ or TDP43‐mutant mice models of amyotrophic lateral sclerosis (Perera et al., 2017, 2021). However, the autophagic potential for rilmenidine has never been explored in *C. elegans* and more importantly, no efforts have been made in any model to associate this upregulation to aging or calorie restriction. We used the mCherry::LGG‐1 reporter strain to quantify autophagosome puncta in the posterior intestinal cells (Gosai et al., 2010; Li et al., 2014; Zhang et al., 2015). We found a significant increase in puncta formation by rilmenidine in a dose‐dependent manner (Figure 3a,b). Notably, prolongevity doses of rilmenidine did not increase autophagy as much as larger doses. To determine whether the increase in autophagy is merely an association or was critical for the longevity effect of rilmenidine, we performed lifespan assays and knocked down essential autophagy genes, *lgg‐1* and *bec‐1*, in WT exposed to rilmenidine or DMSO control. The lifespan extension conferred by rilmenidine was completely abrogated by impairment of autophagy (Figures 3c,d, S3, Table S1). This confirms that autophagy induction is required for rilmenidine to promote longevity (Madeo et al., 2015).

### 2.4 Rilmenidine attenuates poly‐glutamine aggregates

One important hallmark of neurodegenerative diseases is the accumulation of protein aggregates, which should typically be degraded via autophagy. Age‐related protein aggregation and misfolding is evident in routine *C. elegans* aging and precipitate a pronounced, widespread decline in proteostasis. Consistent with this finding, we found that rilmenidine treatments significantly delayed the accumulation of polyQ40::YFP fusion protein aggregates compared to untreated WT (Figure 3e). Besides, this observation attests to our speculation of rilmenidine's function as CRM, since CR too, mediates the reduction in the aggregation of polyQ in *C. elegans* (Matai et al., 2019).

### 2.5 Identification of the conserved imidazoline receptor NISH‐1 in *C. elegans*

Rilmenidine has been identified as a classical imidazoline type 1 receptor (I1R/IRAS/Nischarin) agonist in mammals (Zhang & Abdel‐Rahman, 2006). The amino‐terminal sequence of IRAS displays strong homology to a *C. elegans* protein encoded by the gene *f13e9.1* (Figure S4A) (Alahari et al., 2000). Our comparison of the *C. elegans f13e9.1* full‐length protein sequence to the human ortholog IRAS protein sequence revealed high similarity in the following functional domains; PHOX (PX) domain, leucine‐rich repeats (LRRS), and coiled‐coil (CC) domain (Figure S4A). The PHOX domain entails phosphoinositide‐3‐phosphate‐binding capacity, likely enabling Nischarin, the mammalian form of IRAS, to be anchored to the intracellular surface of the plasma membrane (Piletz, 2003). The coiled‐coil domain is the predicted imidazoline‐binding site (Sun et al., 2007) (Figure S4A). Thus, we renamed the *f13e9.1* gene to *nish‐1* and will refer to it as *nish‐1* throughout this study.

**FIGURE 4:**
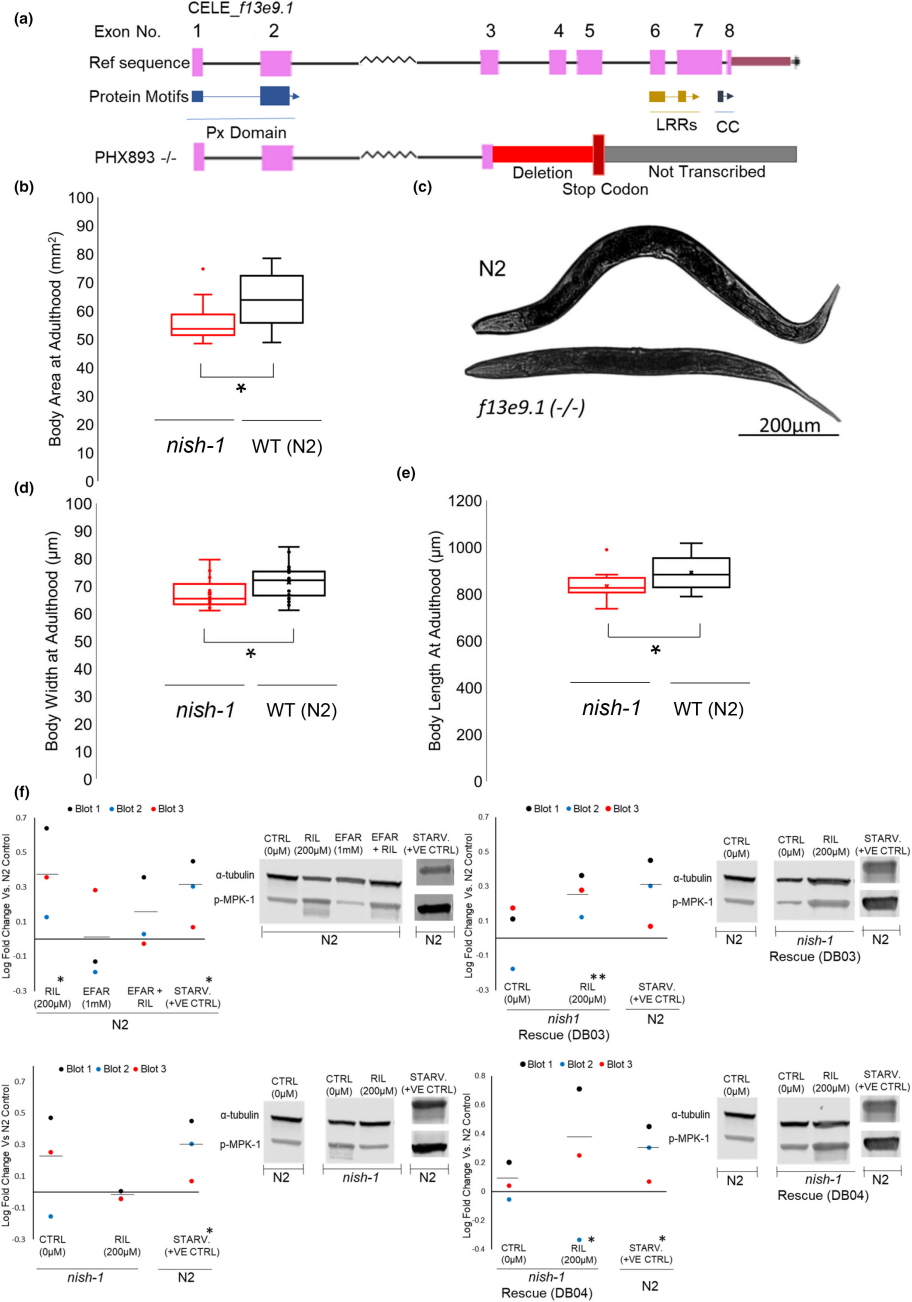
Characterization of NISH‐1. (a) Schematic diagram illustrating exons of *nish‐1* gene, alongside translated protein motifs and the deleted regions in the homozygous *nish‐1* mutant. (b–e) *nish‐1* mutants exhibit reductions in body size compared to WT at day 1 of adulthood as shown by body area (defined as width x length). A two‐tailed *t*‐test was used for analysis; *p < 0.05. Representative pictures of WT and *nish‐1* mutant captured in brightfield at 10X objective on a Zeiss Axio Observer following paralysis in 20 mM tetramisole. Animal heads are left. Scale bar: 200 μm. (f) Western blots showing MPK‐1 phosphorylation in WT, *nish‐1* mutants, or *nish‐1* rescue transgenic animals, in response to 24 h pharmacologic intervention. α‐tubulin was used as the loading control. Rilmenidine significantly increased MPK‐1 phosphorylation *(%CV vs FC = >1.5), however, neither efaroxan alone nor rilmenidine and efaroxan in combination significantly increased MPK‐1 phosphorylation, n/s (%CV vs FC = <1.5). Rilmenidine failed to reproducibly increase phosphorylation of MPK‐1 in *nish‐1* mutants. n/s (%CV vs FC = <1.5). Transgenic rescue strains significantly increased MPK‐1 phosphorylation upon rilmenidine treatment. **(%CV vs FC = >2 in PHX945) and (%CV vs FC = >1.5 in PHX946). Data are quantified as log fold densitometric ratio change relative to α‐tubulin. Data are then expressed as log fold change compared to DMSO vehicle control. Coefficient of variation (%CV) defined as the percent standard deviation: mean ratio; the significant difference in groups if percent fold change is ×1.5* greater or ×2** than % CV.

To examine the role of *nish‐1* as a functional ortholog of Nischarin that could mediate rilmenidine cell signaling, we generated a loss‐of‐function mutant *nish‐1 (syb767)* by CRISPR/Cas9 genome editing. A deletion of 873 bp was initiated at the third exon and a stop codon was introduced to generate a potential null allele (see Materials and Methods for details). This resulted in truncated NISH‐1 protein, containing only the PHOX domain but abolishing LRRs and the coiled‐coil domain (Figure 4a). We validated the deletion in the *nish‐1* mutant by genotyping (Figure S4B).

Murine nischarin mutants are smaller than their wild‐type counterparts (Crompton et al., 2017; Dong et al., 2017; Zhang et al., 2013). Therefore, for the functional characterization of the *nish‐1* mutant, we first measured their body size. Day 1 adult *C. elegans nish‐1* mutants were 5.44% smaller than WT (*p*‐value <0.05; Figure 4b–e). To exclude slower development as the reason for size defects seen in *nish‐1* mutants, we assessed the development rate of animals via the categorical staging of vulval development (Ludewig et al., 2017). Both WT and *nish‐1* mutant developed at a similar rate and had reached full vulval development within 48 h (Figure S4C). Also, *nish‐1* mutants did not exhibit any reproductive developmental abnormalities.

### 2.6 Rilmenidine requires *nish‐1* to extend life span, improve markers of health span, and stress response

As rilmenidine has never been studied in *C. elegans*, thus it was essential to establish that rilmenidine is indeed bioactive in *C. elegans* and that lifespan extension and cellular effects of rilmenidine in *C. elegans* is not merely a hormetic response, but indeed, conferred via interaction with an endogenous imidazoline‐binding site (IBS) in *C. elegans*.

Studies have shown that the interaction of rilmenidine with an I1‐imidazoline receptor leads to activation of phosphatidylcholine‐specific phospholipase C (PC‐PLC) and subsequent accumulation of the second messenger diacylglyceride (DAG) from phosphatidylcholine, and the release of phosphocholine. This causes downstream activation of MAPK/ERK1/2, with phosphorylation of ERK1/2 being a common read‐out for the pathway (Zhang & Abdel‐Rahman, 2006). *C. elegans* contains only one ERK gene (*mpk‐1*), rendering it the more facile and reliable system in the pathway to measure compared to the six isoforms of PLC. The activation of MPK‐1 was measured, by way of pMPK‐1 to α‐tubulin immunoreactivity, in response to 24 h treatment with rilmenidine at varying concentrations (200, 300, and 400 μM) as per (Chen et al., 2008; Villanueva‐Chimal et al., 2017) (Figure S4D). Rilmenidine increased MPK‐1 phosphorylation the strongest at the concentration of 200 μM; the same concentration that elicits the greatest prolongevity effect. It is not inconceivable that hormetic stress from rilmenidine administration may have upregulated ERK activity entirely independent from any imidazoline receptor activity, however, 24 h treatment with efaroxan, an established selective I1‐imidazoline receptor antagonist, abolished ERK activation by 200 μM rilmenidine. This suggests rilmenidine treatment most likely interacts with an endogenous imidazoline‐binding site in *C. elegans* to elicit increases in ERK activity (Figure 4c).

**FIGURE 5:**
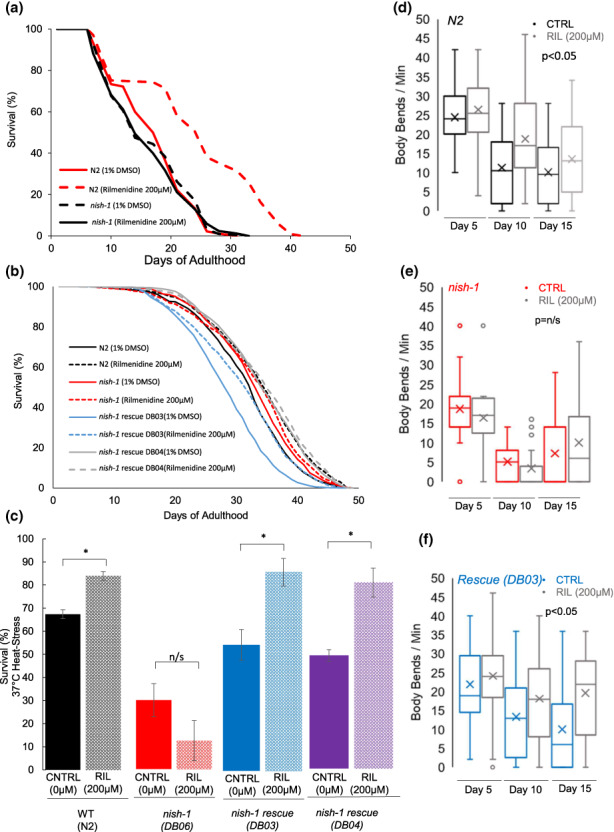
*nish‐1* is required for an extended lifespan and better health span. (a) Survival curve showing lifespan extension by rilmenidine in WT, but not in *nish‐1* mutant (Statistics and raw data are in Table S1). (b) Rescue of *nish‐1* enabled extension in lifespan by 200 μM rilmenidine in *nish‐1* mutant. DB06 (=PHX893) is a *nish‐1* knockout mutant and DB03 (=PHX945) and DB04 (=PHX946) are two rescue lines expressing wild‐type *nish‐1* gene copies. The difference in control‐treated lifespan between DB03 and DB04 might be the random site of integration of the rescue transgene. Quantitative data and statistical analyses for the representative experiments are included in Table S1. (c) Thermotolerance: Rilmenidine increases the percentage survival of WT exposed to 37°C for 3 h and approximately 20 h recovery period, dependent on *nish‐1*. Bar graph represents mean % survival ± SEM from three independent trials of at least 150 animals per strain and/or condition. *p < 0.05 (one‐way ANOVA with Tukey post hoc comparisons). (d–f) Quantified data of body bends representing motility deterioration with age in WT, *nish‐1* mutants, and two *nish‐1* rescue mutants DB03 and DB04 in the presence or absence of rilmenidine. Rilmenidine at 200 μM significantly reduced age‐related motility deterioration in WT and *nish‐1* rescue strains, but not in *nish‐1* mutants. Data are represented as a pooled mean of 10 animals per time point and condition/genotype repeated over three independent trials overlaid on a box plot representing quartiles. Error bars, upper: Q3 + 1.5*IQR; minimum: Q1–1.5*IQR (adj. *p* < 0.05; two‐way ANOVA and Tukey post hoc).

To determine if *nish‐1* is required for the lifespan extension by rilmenidine, adult WT and *nish‐1*‐mutant *C. elegans* were incubated from day 1 adulthood with rilmenidine (200 μM) and lifespan assay was performed. We found that the lifespan‐extending effects of rilmenidine were abolished when *nish‐1* was deleted. Rilmenidine‐treated *nish‐1* mutants lived as long as WT and *nish‐1* mutants without rilmenidine (Figure 5a). Critically, rescuing the *nish‐1* receptor reinstated the increase in lifespan upon treatment with rilmenidine (Figure 5b).

*Caenorhabditis elegans* exhibit a comparable age‐associated deterioration in muscle function to humans (Glenn et al., 2004; Herndon et al., 2002). Moreover, studies have shown that the rate of age‐related decline in body movement is a good predictor of lifespan and that long‐lived *C. elegans* mutants tend to exhibit prolonged locomotory capacity (Herndon et al., 2002; Huang et al., 2004; Newell Stamper et al., 2018). Importantly, calorie restriction in *C. elegans* preserves body movement capacity with age, and putative CRMs are able to replicate this phenomena (Calvert et al., 2016; Onken & Driscoll, 2010a, 2010b). Using the common assay of sigmoidal motility, we measured locomotory capacity in WT exposed to 200 μM rilmenidine. Rilmenidine significantly ameliorated deterioration in WT animal motility over the three time points, days 5, 10, and 15. Moreover, the supplementation of *nish‐1‐*mutant animals with 200 μM rilmenidine from day 1 failed to defer declines in motility. However, rescue of *nish‐1* mutants was able to reinstate the preservation of motility into age by rilmenidine as observed in WT animals (Figure 5d–f).

Longevity is associated with better stress survival (Zhou et al., 2011). Evidence also suggests, calorie restriction may delay aging phenotypes through a preservation of homeostatic flexibility and improved resilience. Thus, for rilmenidine to be considered as a viable geroprotector, it should improve health span and ideally, to qualify as a CRM, and improve stress resistance. Here, we determined the thermotolerance of animals exposed to rilmenidine. Loss of *nish‐1* did not affect resilience to heat. Rilmenidine treatment improved the thermotolerance at 37°C of WT animals but not *nish‐1* mutants (Figure 5c). Transgenic rescuing of the NISH‐1 receptor in *nish‐1* mutants restored rilmenidine's resistance to heat stress (Figure 5c).

Taken together, this provides evidence that not only is *nish‐1* required for rilmenidine to extend lifespan, but it is also essential to defer aging phenotypes and improve stress resistance in *C. elegans*.

### 2.7 Rilmenidine produces longevity‐associated gene expression effects in mouse tissues

**FIGURE 6:**
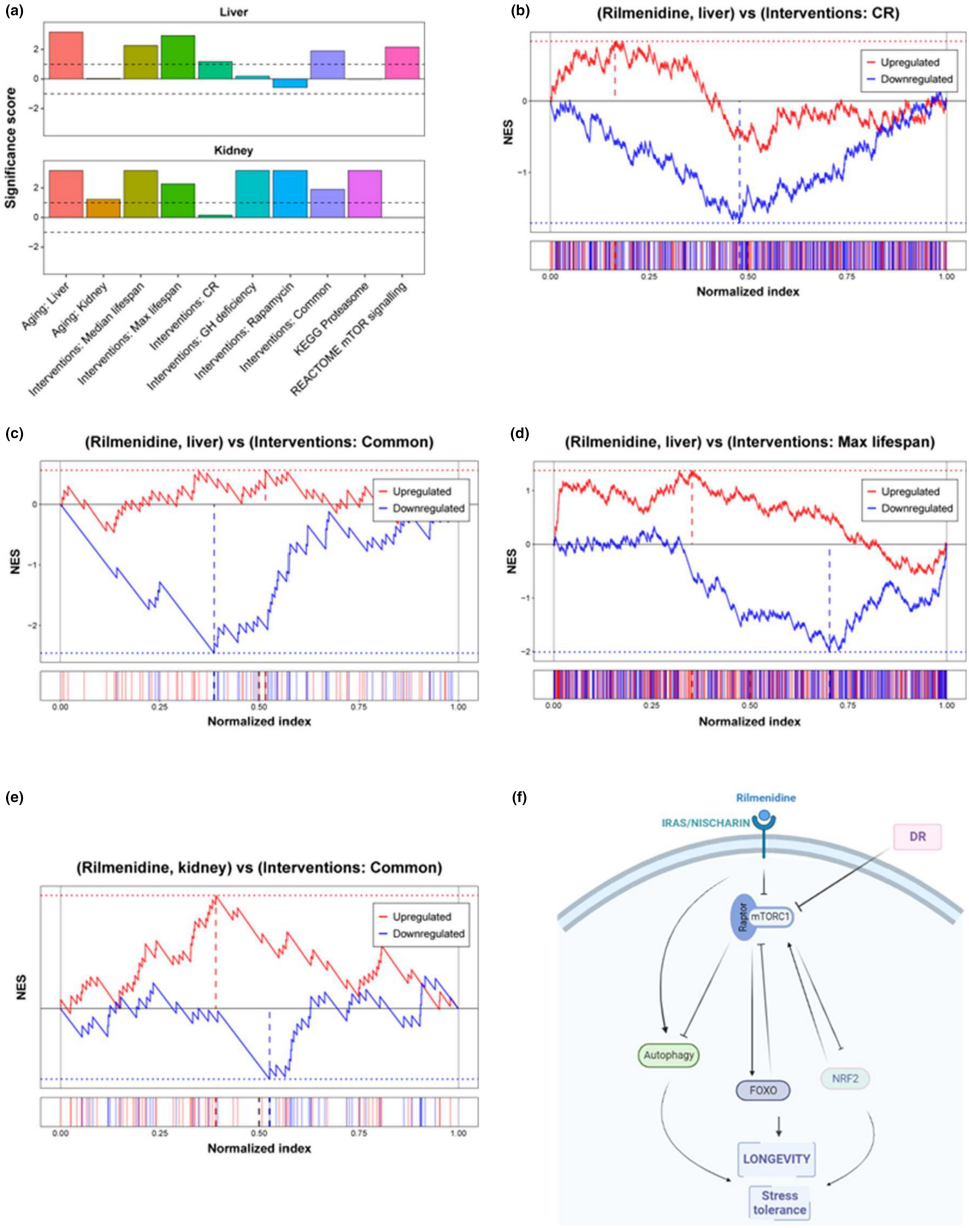
Rilmenidine treatment in mice induces gene expression changes associated with a lifespan‐extending effect. (a) Association of the liver (top) and kidney (bottom) responses to rilmenidine with signatures of aging (left), lifespan extension (middle), and longevity‐related intracellular processes (right). The significance score was calculated as log_10_(adjusted *p*‐value) corrected by a sign of regulation. Dotted lines represent the FDR threshold of 0.1. (b–e) GSEA enrichment plots with running normalized enrichment scores (top) and distributions (bottom) of selected up‐ (red) and downregulated (blue) gene signatures of lifespan‐extending interventions among genes perturbed by rilmenidine in the liver (b–d) and kidney (e). Perturbed genes were sorted based on the log_10_(*p*‐value) of their differential expression between control and rilmenidine‐treated samples corrected by a sign of regulation. The resulting index was divided by the number of genes in the dataset. Dotted lines represent NES calculated for up‐ (red) and downregulated (blue) gene signatures. (f) Working model representing possible prolongevity signalling by rilmenidine to extend life span in *C. elegans*. NES, Normalized enrichment score; CR, Caloric restriction; GH, Growth hormone; mTOR, mammalian target of rapamycin.

To test if the observed beneficial effects of rilmenidine are mirrored in mammals, we performed RNA‐seq analysis of liver and kidney samples from young UM‐HET3 male mice subjected to rilmenidine for 1 month, together with the corresponding controls. We identified transcriptomic effects of the drug independently for each tissue and compared them with biomarkers of lifespan‐extending interventions and aging, using a gene set enrichment analysis (GSEA)‐based approach (Figure 6a) (Tyshkovskiy et al., 2019). Gene expression signatures of lifespan extension reflect a liver response to individual interventions, such as CR, rapamycin and mutations leading to growth hormone (GH) deficiency; gene expression changes shared by different longevity interventions; and genes, which expression is quantitatively associated with the effect on maximum and median lifespan (Tyshkovskiy et al., 2019). Aging signatures correspond to murine age‐related gene expression changes in the liver and kidney, according to the Tabula Muris Consortium (Schaum et al., 2020).

Interestingly, changes induced by rilmenidine in both liver and kidney were positively associated with the majority of longevity signatures, including biomarkers of median and maximum lifespan extension (adjusted *p*‐value <2 × 10^−3^ for both liver and kidney), CR (adjusted *p*‐value = 0.066 for liver), rapamycin (adjusted *p*‐value = 6.7 × 10^−4^ for kidney), and common signatures of longevity interventions (adjusted *p*‐value <0.013 for both liver and kidney). In other words, genes up‐ and downregulated in long‐lived mice were also changed in the same direction after rilmenidine administration (Figure 6b–e). Remarkably, we also observed a significant positive association of rilmenidine effect with biomarkers of aging (median adjusted *p*‐value = 0.03), which may be partly due to the age‐related downregulation of insulin and IGF‐1 signaling (López‐Otín et al., 2013).

To understand if rilmenidine in mice regulates cellular functions similar to those identified in *C. elegans*, we also performed functional GSEA. This method allowed us to test if genes involved in certain pathways, based on REACTOME, KEGG and GO BP annotation, were enriched among those affected by the compound. We observed significant enrichment of genes involved in mTOR signaling and proteolysis among genes upregulated by rilmenidine in murine liver and kidney, respectively (adjusted *p*‐value <7 × 10^−3^) (Figure 6a). These results suggest that longevity‐associated molecular mechanisms induced by rilmenidine in *C. elegans* are reproduced in mammals, pointing to a conserved geroprotective effect and downstream signaling (Figure 6f).

## 3 DISCUSSION

Challenges with humans' long‐term CR routines raise an unmet need to explore pharmacologic interventions like CRMs. These may provide an alternative to CR, conferring the same longevity benefits without the challenges or side effects of low‐calorie diets. However, studies detailing the therapeutic efficacy of CRMs when administered at geriatric stages are rarely performed (Ingram & Roth, 2015). Therefore, identifying drugs that can mirror the benefits of long‐term calorie restriction even when the administration is initiated at late stages of life are of great interest.

Our study illustrates that rilmenidine treatment elicits lifespan extension in *C. elegans* even when initiated in later life (commencing day 12 of adulthood) after the development of age‐associated phenotypes. This is furthered by our work showing rilmenidine can delay the onset of frailty and proteostatic collapse, both phenotypes of aging. Specifically, rilmenidine was able to defer locomotory decline in aging animals while also delaying the rate of polyQ aggregation. Clinical evidence of imidazoline agents including rilmenidine being tolerable and safe in elderly populations while also ineffective at changing body weight, supports its potential implementation later in life and in murine models (Kirkendall, 1986; Martin et al., 2005; Nowak et al., 2005; Rose et al., 2010).

The clinical target of rilmenidine, human IRAS, was hitherto, undiscovered in *C. elegans*. We identified a putative *C. elegans* ortholog using a bioinformatic approach showing significant protein sequence alignment between IRAS and the uncharacterized F13E9.1. Furthermore, an outcrossed mutant strain encoding a homozygous deletion of *f13e9.1* exons 3–8, generated in this article, demonstrated similar body size phenotype to IRAS‐knockout mice.

However, it remained unknown whether, in *C. elegans*, rilmenidine exhibited bioactivity characteristic of imidazoline receptor activation and whether this specific bioactivity derived its prolongevity effect. Increases in ERK phosphorylation are consistently observed following rilmenidine treatment in cell lines, mediated through alterations in PKC. Indeed, in *C. elegans*, rilmenidine increased ERK phosphorylation in a characteristic biphasic response (Piletz et al., 2000). Since extracellular stimuli such as growth factors, cytokines, mitogens, hormones, and oxidative or heat stress are known to increase ERK phosphorylation (Mebratu & Tesfaigzi, 2009), it is possible rilmenidine administration caused a similar stimulus and thus upregulated ERK activity independent of imidazoline receptor activity. However, animals co‐incubated with rilmenidine and imidazoline receptor antagonist efaroxan abrogated increases in ERK phosphorylation. Furthermore, *f13e9.1* (*nish‐1*)‐mutant strains similarly abrogated rilmenidine‐induced ERK phosphorylation, suggesting not only that ERK phosphorylation was dependent on an available imidazoline‐binding site, but also that these were dependent on NISH‐1 expression.

Such activity was mirrored in our lifespan and healthspan studies. Both *nish‐1* mutants and efaroxan treatment abrogated the prolongevity effect of rilmenidine, providing evidence that the interaction of rilmenidine on an IBS, likely found on NISH‐1, drives the geroprotective properties of rilmenidine. Meanwhile, *nish‐1* was also essential for and deferred locomotory decline in *C. elegans*: a key indicator of health span. Our work also shows that rilmenidine was able to improve thermotolerance in *C. elegans* again dependent on *nish‐1¸* strengthening the importance of *nish‐1 in* rilmenidine‐induced longevity.

The upregulation of autophagy genes appears to comprise some of the heat shock response (Kumsta et al., 2017). Furthermore, it is suggested that CR exerts its benefit through the activation of a heat‐shock response that increases autophagy (Yang et al., 2016). The induction of autophagy by rilmenidine, as demonstrated in this article, may drive an improvement of nematode thermotolerance and mediate improved protein homeostasis. In vitro and murine work has confirmed rilmenidine can induce autophagy, however, this has never been conceived in *C. elegans* or in the aging arena (Perera et al. 2017; Rose et al., 2010). Accordingly, rilmenidine increased autophagy in a dose‐dependent manner. Not only did rilmenidine increase autophagic induction in *C. elegans*, but also the prolongevity benefit of rilmenidine was dependent on key autophagy genes. This pattern is mirrored in *eat‐2* mutant nematodes who require intact autophagy to extend lifespan (Hansen et al., 2008). This is important because prolongevity doses of rilmenidine did not increase autophagy as much as larger toxic doses. While plenty of evidence has demonstrated autophagy induction to increase a multitude of aging markers, it represents a sharp double‐edged sword, whereby overactive autophagy may actually precipitate cellular pathology (Benedetto et al., 2019; Benedetto & Gems, 2019; Kubli & Gustafsson, 2014; Qiu et al., 2018; Rubinsztein et al., 2011).

Interactions between rilmenidine and CR in the form of reduced swallowing capacity (*eat‐2*) demonstrated the inability of rilmenidine to extend lifespan in an additive manner; instead, rilmenidine doses that normally benefit wild‐type were actually deleterious to *eat‐2*, perhaps through a hyper‐induction of CR signaling. Nonetheless, there are several CR regimes in *C. elegans*, and thus interaction studies of rilmenidine with other CR regimes and across different levels of CR are now warranted.

As previously highlighted, two key nutrient responsive gene products, AMPK and TOR, have been considered “master regulators” of dietary restriction. The ability of potential CRMs to affect the activity of these pathways specifically may offer a more orthogonal approach to CR mimicry without superfluously auxiliary effects.

Downstream genetic signalling pathways of CR, DAF‐16 and TOR, contributed to the ability of rilmenidine to extend lifespan, although AMPK was not critical to the prolongevity effect. Rilmenidine lifespan extension was entirely dependent on normalized TOR function (whether that be pharmaceutically or genetically downregulated), implying that rilmenidine signalling likely converged on TOR to elicit most of its geroprotective properties. How rilmenidine might interact with TOR remains to be seen. It has been tentatively proposed that in vitro, rilmenidine upregulates autophagy by reducing intracellular cAMP levels, prohibiting IP3‐mediated Ca^2+^ release from the ER (Renna et al., 2010). Indeed, it is mechanistically proven that in vitro, rilmenidine can reduce both basal and forskolin‐stimulated cAMP levels (Greney et al., 2000). Thus, it is logical to hypothesize that rilmenidine may increase autophagy via downregulation of cAMP signalling in *C. elegans* as well, effectively inducing autophagy independent of mTOR. Alternatively, given that rilmenidine also required *daf‐16* to elicit lifespan extension, it is possible that rilmenidine causes TOR inhibition leading to *daf‐16‐*induced transcription of heat‐shock genes (Robida‐Stubbs et al., 2012). The necessity of *ce*TOR for rilmenidine‐induced longevity is then reconciled with the requirement of alternative pathways that depends on autophagy. For example, rilmenidine may not only clear cellular waste, but may also help in the generation of new raw material for protein synthesis that requires functioning ceTOR. This provides a potential mechanism of rilmenidine‐induced geroprotection and thermotolerance. This would differentiate rilmenidine from rapamycin in its mechanism, which is *daf‐16* independent, owing to its interaction with TORC2 (Robida‐Stubbs et al., 2012). Chronic administration of rapamycin is associated with activation of mTORC2 which supposedly causes detrimental effects on metabolism, including hyperglycemia, hyperlipidemia, and insulin resistance in mice, and thus, if rilmenidine can precisely downregulate mTORC1 signalling, without affecting mTORC2, it may present a more tolerable and durable CRM than rapamycin (Lamming et al., 2012; Robida‐Stubbs et al., 2012).

In summary, this research presents a novel case for rilmenidine to be considered a potential calorie restriction mimetic through its prolongevity and health preserving effects, increased stress resistance, and increased autophagy. Alongside being a clinically approved antihypertensive drug, rilmenidine improves plasma lipid and blood glucose in patients with hypertension and metabolic syndrome (Luca et al., 2000; Reid, 2001). Given its contribution toward lowering blood glucose and increasing insulin sensitivity, rilmenidine may have an antidiabetic function. Since I1R and I3R do not share similar ligand‐binding sites and I3R is a regulator of insulin secretion, the potential interaction of rilmenidine with the I3type imidazoline receptor might explain its effects on improved insulin resistance and, thus, glucose levels (Bousquet et al., 2020). As rilmenidine improves glucose tolerance, it might eventually be put in the insulin sensitizer class of CRMs (Bousquet et al., 2020).

Rilmenidine has a good clinical profile and chronic administration is rarely a problem in hypertensives (Reid, 2000); it becomes feasible for rilmenidine to be repositioned against insulin resistance, metabolic syndrome, and polyglutamine diseases. Moreover, conservation of findings between *C. elegans*, mice, and human cell culture, such as induction of autophagy, points toward the more considerable potential for translating the longevity benefits to humans. In conclusion, rilmenidine is a new addition to the list of potential CRMs that could be viable therapeutic interventions administered later in life, and thus, warrants further examinations.

## AUTHOR CONTRIBUTIONS

All authors participated in analyzing and interpreting the data. DFB, JPdM, and CYE designed the experiments. DFB and CYE performed manual lifespan assays, AG did the food uptake experiment and CS performed automated lifespan machine assays. AT and VNG performed mice experiments and analyses. All other experiments were performed by DFB. DFB, AG, CWB, CYE, and JPdM wrote the article in consultation with the other authors.

## CONFLICT OF INTEREST

JPM is an advisor/consultant for the Longevity Vision Fund, NOVOS, YouthBio Therapeutics, and the founder of Magellan Science Ltd, a company providing consulting services in longevity science. CYE is a co‐founder of AVEA Life AG and an advisor for Maximon AG Longevity Startup builder. The other authors have no competing interests to declare. Correspondence should be addressed to CYE and JPdM.

## Supporting information


Table S1

Table S2

Table S3

Appendix S1

## Data Availability

RNA‐seq data are available in GEO (liver data in GSE131868; kidney data in GSE206982). All the other data are provided in Supplementary Tables in this study.
