# Supplementary material for: Rilmenidine extends lifespan and healthspan in Caenorhabditis elegans via a nischarin I1‐imidazoline receptor
Source: Aging Cell. 2023 Jan 20;22(2):e13774. doi: 10.1111/acel.13774 (PMC9924948; doi:10.1111/acel.13774)
Supplement: Supplementary file 4 — Appendix S1 [file ACEL-22-e13774-s001.pdf]

Supplementary Material:

Rilmenidine extends lifespan and healthspan in *C. elegans* via a nischarin I1-imidazoline receptor

Dominic F. Bennett[1§], Anita Goyala[2§], Cyril Statzer[2], Charles W. Beckett[1], Alexander Tyshkovskiy[3,4], Vadim N. Gladyshev[3], Collin Y. Ewald[2*], and João Pedro de Magalhães[1,5*]

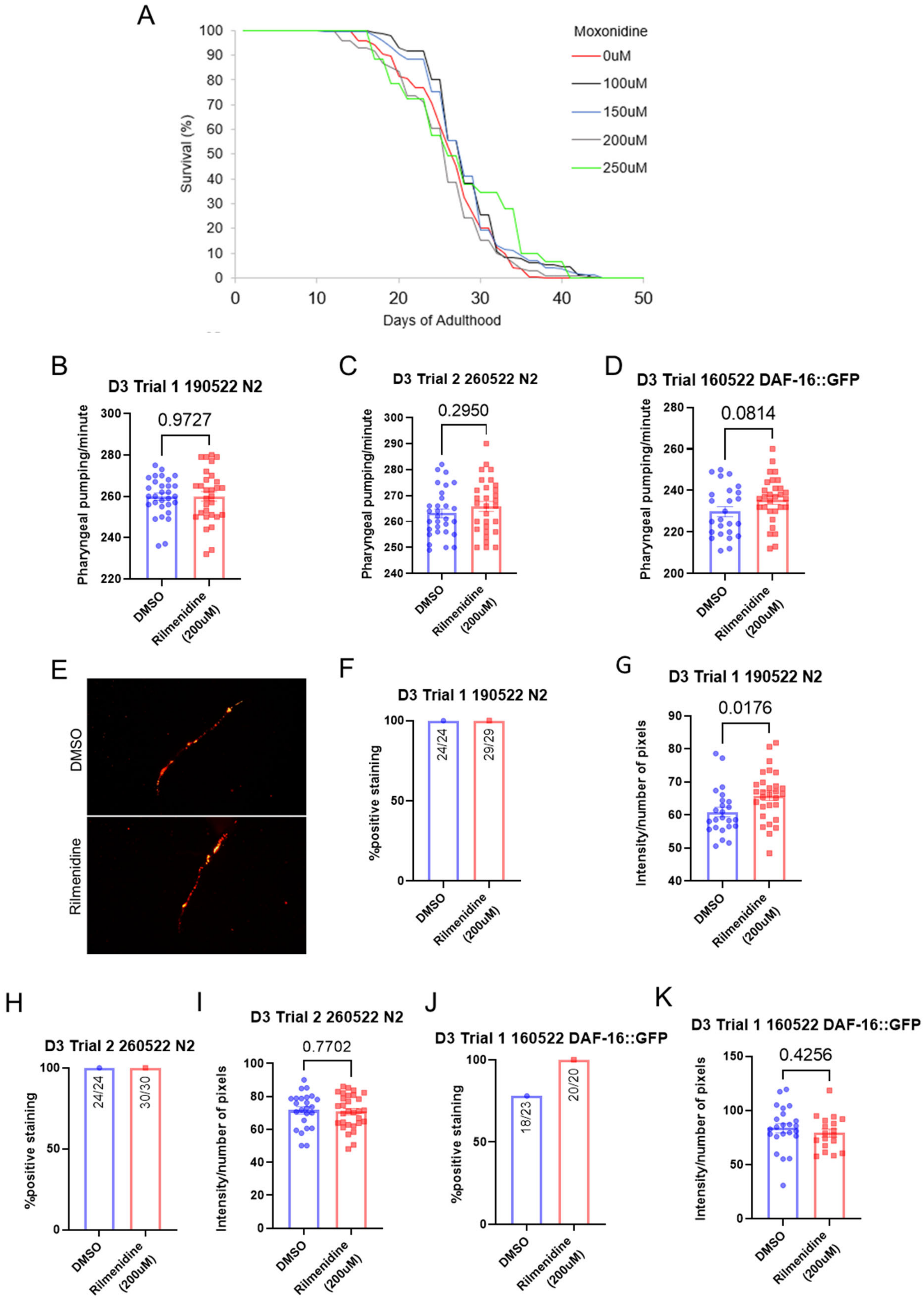
A
Moxonidine
0uM
100uM
150uM
200uM
250uM
Survival (%)
Days of Adulthood
B
D3 Trial 1 190522 N2
0.9727
Pharyngeal pumping/minute
DMSO
Rilmenidine (200uM)
C
D3 Trial 2 260522 N2
0.2950
D
D3 Trial 160522 DAF-16::GFP
0.0814
E
DMSO
Rilmenidine
F
D3 Trial 1 190522 N2
%positive staining
24/24
29/29
G
D3 Trial 1 190522 N2
0.0176
Intensity/number of pixels
H
D3 Trial 2 260522 N2
24/24
30/30
I
D3 Trial 2 260522 N2
0.7702
J
D3 Trial 1 160522 DAF-16::GFP
18/23
20/20
K
D3 Trial 1 160522 DAF-16::GFP
0.4256

Figure S1: A) Survival curve of WT treated with moxonidine at different concentrations. Statistics and raw data are in Supplementary Table 1. **B-D)** Three biological replicates showing **pharyngeal pumping rate** in rilmenidine treated WT **(B, C),** and DAF-16::GFP **(D),** as compared to DMSO. Quantified data shown as mean + SEM. *P*-value determined by two-tailed Welch's *t*-test. **E-K)** Three biological replicates for **food uptake** by rilmenidine treated WT **(E-I)** and DAF-16::GFP **(J, K)** animals, compared to DMSO. E) Representative images for animals showing fluorescent beads in the intestine. F, H, J) Percentage of total animals positive for fluorescence from beads uptake. Number of animals is indicated in the bar. G, I, K) Fluorescence Intensity per number of pixels was measured to quantify uptake of beads. Quantified data shown as mean + SEM. *P*-value determined by two-tailed Welch's *t*-test.

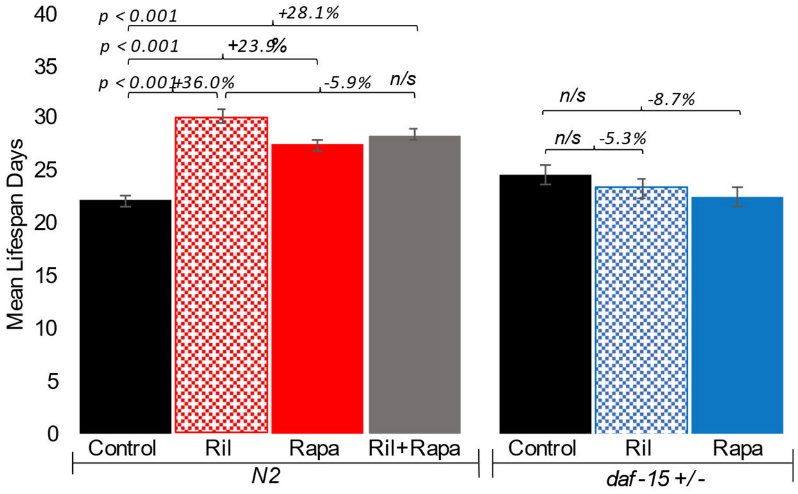

Figure S2: Related to figure 2B, C. Quantified data for mean lifespan (days) and % change in lifespan upon rilmenidine administration in *daf-15(m81/+)* mutants and rapamycin-treated WT. Error bars represent ± SEM; adjusted p-value was derived from log-rank test and Bonferroni correction. See Supplementary Table 1 for details.

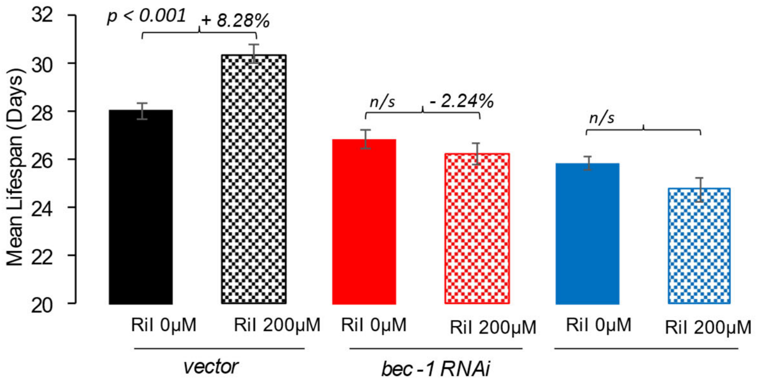

Figure S3: Related to figure 3C, D. Quantified data for mean lifespan (days) and % change in survival of WT upon knockdown of autophagy genes, *bec-1* and *lgg-1* alongside rilmenidine treatment. Rilmenidine significantly increased lifespan by 8.29% in WT animals fed HT115 *E. coli* expressing an empty vector (p<0.001), however, failed to significantly affect lifespan in animals fed HT115 *E. coli* expressing *bec-1* or *lgg-1* dsRNA; n/s = p>0.05. See Supplementary Table 1 for details.

A

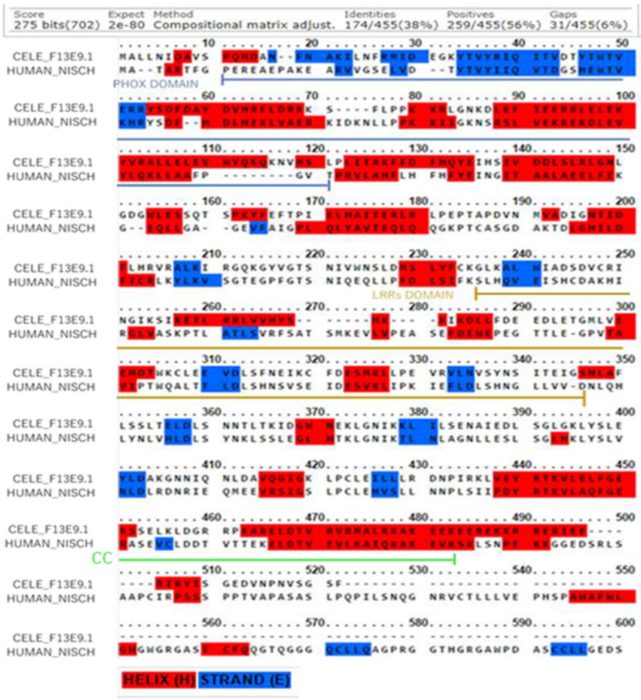
Score
275 bits(702)
Expect
2e-80
Method
Compositional matrix adjust.
Identities
174/455(38%)
Positives
259/455(56%)
Gaps
31/455(6%)
CELE_F13E9.1
HUMAN_NISCH
PHOX DOMAIN
LRRs DOMAIN
CC
HELIX (H)
STRAND (E)

B

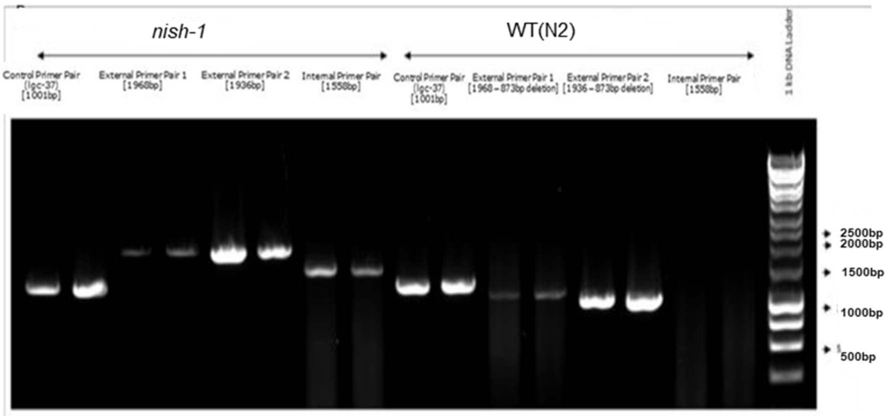
nish-1
WT(N2)
Control Primer Pair (lgc-37) [1001bp]
External Primer Pair 1 [1968bp]
External Primer Pair 2 [1936bp]
Internal Primer Pair [1558bp]
External Primer Pair 1 [1968 – 873bp deletion]
External Primer Pair 2 [1936 – 873bp deletion]
1 kb DNA Ladder
2500bp
2000bp
1500bp
1000bp
500bp

C

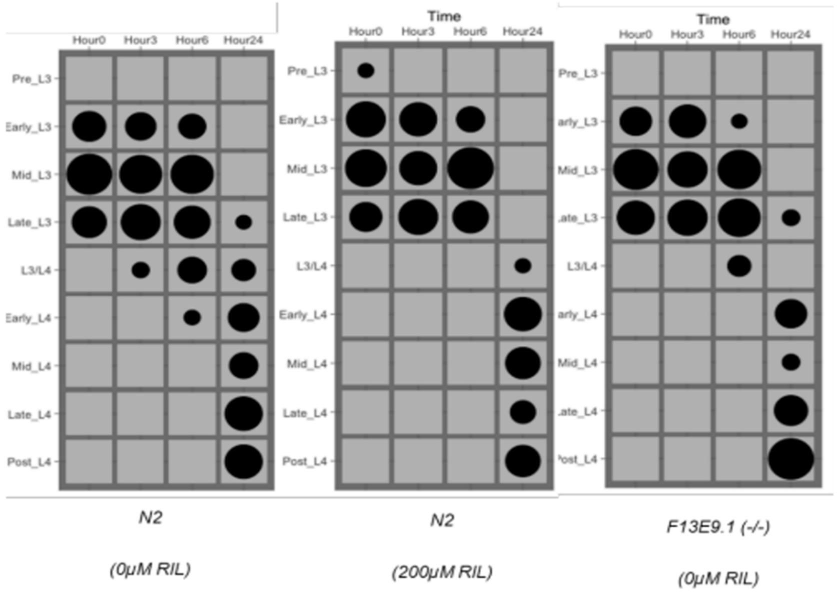
Time
Hour0
Hour3
Hour6
Hour24
Pre_L3
Early_L3
Mid_L3
Late_L3
L3/L4
Early_L4
Mid_L4
Late_L4
Post_L4
N2
(0μM RIL)
N2
(200μM RIL)
F13E9.1 (-/-)
(0μM RIL)

Figure S4. A) BLASTp alignment of *C. elegans* NISH-1 protein sequence (CELE_F13E9.1 – UniProtKB-K8F807) against truncated human NISCHARIN isoform 3 (HUMAN_NISCH – UniProtKB-Q9Y2I1-3) revealing 38% sequence similarity, with conserved secondary structure elements like alpha helices shown in red and beta-strand in blue. Functional protein motifs like PHOX (Px) domains, LRRs, and coiled-coil domains are underlined. **B)** Endpoint single *C. elegans* PCR genotyping of WT and *nish-1* deletion mutant following x4 outcrossing. Using oligonucleotides flanking the deletion site (external primer pairs) generated either a 1968 bp or a 1936 bp amplicon depending on oligonucleotide pair in WT or a correspondingly 873 bp reduced amplicon size in the genomic DNA of homozygous *nish-1* mutants. Likewise, an oligonucleotide pair targeted to sequences within the CRISPR deletion site produced a 1558 bp amplicon in WT but no amplicon in homozygous *nish-1* mutants. Control amplifications were conducted on the *lgc-37* gene coded on chromosome III. Primer sequences are in Supplementary Table 2. **C)** *nish-1* mutants and rilmenidine-fed WT do not exhibit delayed vulval development from the L3 stage. In the chart, developmental stages are listed on the Y-axis, and the number of hours after a 24h exposure to food from L1 is plotted on the x-axis. The areas of the circles in the chart reflect the percentage of the population at each stage of development; n≥30 from 3 independent pooled trials for each time point. Statistical difference established by way of 2-way ANOVA wherein a mean development score could be ascertained through pre-L3 corresponding to a score of 0, Early L3 is 1 and Mid L3 a 3 etc. No groups displayed any significant difference in development rate.

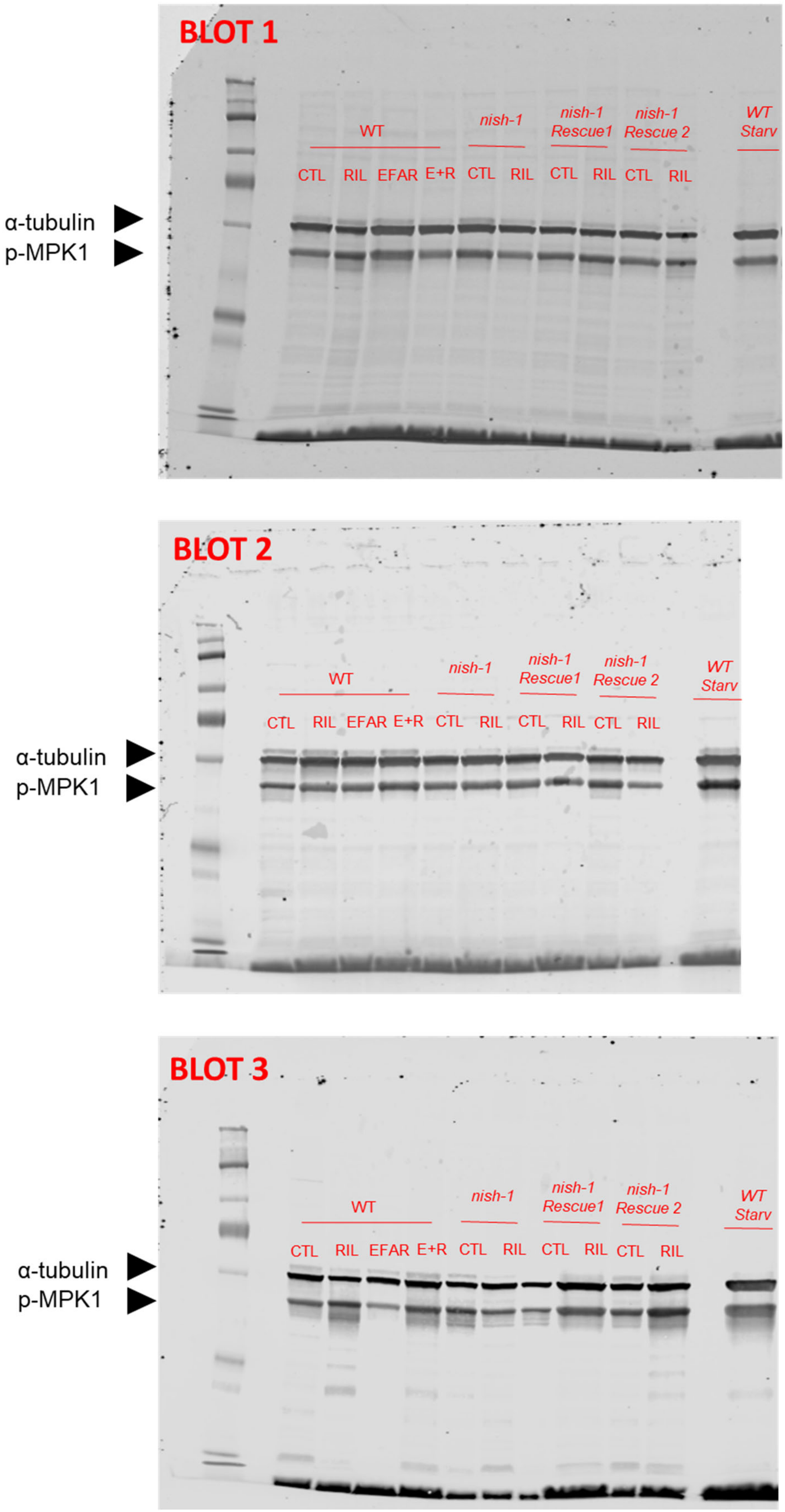

Figure S5. Uncropped western blots relating to Fig 4F.

Supplementary Table 1: Lifespan summary and details.

Supplementary Table 2: Primer sequences.

Supplementary Table 3: GSEA output.

Materials and Methods

C. elegans Strains

All strains were maintained on OP50 *E. coli* seeded NGM agar supplemented with 50 IU/mL penicillin (Sigma #P4333), 50 µg/ml streptomycin (Sigma #P4333), and 50 IU/mL Nystatin (Sigma #1638) to prevent contamination (Stiernagle 2006). The strains used in the study are: WT (N2 Bristol), PHX893 (=DB06) *nish-1(syb767),* PHX117 *sybIs62* [*f13e9.1*::EGFP::3xFLAG, *unc-119*(+) + P*myo-2*::mCherry], PHX116 *sybIs62* [*f13e9.1*::EGFP::3xFLAG, *unc-119*(+) + P*myo-2*::mCherry], PHX945 (=DB03) and PHX946 (=DB04) are two independent integrated lines of *f13e9.1 (syb767)* IV; *sybIs62* [*f13e9.1*::EGFP::3xFLAG, *unc-119*(+) + P*myo-2*::mCherry], VK1093 *vkEx1093* [P*nhx-2*::mCherry::*lgg-1*], AM141 *rmIs133* [P*unc-54*::Q40::YFP], GR1307 *daf-16(mgDf50)* I; DA1116 *eat-2(ad1116)* II; LD1057 *skn-1(tm3411)* (Ewald et al. 2015) IV, DR412 *daf-15(m81)/unc-24(e138)* IV; TG38 *aak-2(gt33)* X, and TJ356 zIs356 [*daf-16*p::DAF-16a/b::GFP + *rol-6*(*su1006*)].

The transgenic strains PHX893 (=DB06) *nish-1(syb767),* PHX117 *sybIs62* [*f13e9.1*::EGFP::3xFLAG, *unc-119*(+) + P*myo-2*::mCherry], and PHX116 *sybIs62* [*f13e9.1*::EGFP::3xFLAG, *unc-119*(+) + P*myo-2*::mCherry] were generated by SunyBiotech (Fuzhou, Fu Jian Province, China). In PHX893 (=DB06) *nish-1(syb767),* the 3rd exon, 4th exon, and part of 5th exon, was deleted and replaced by a splice site and stop codon that terminates transcription and resulted in 873 bp homozygous deletion in the *nish-1* gene. This strain was subsequently outcrossed four times against WT background using PCR screening at each stage, and the final outcrossed strain was confirmed by sequencing. The details of the primers are documented in Supplementary Table 2. PHX117 and PHX116 contain integrated full-length rescue of the *nish-1* locus within the fosmid generated in the WT background. Specifically, the integrated rescue contained the WRM0616C_F02(pRedFlp-Hgr) (F13E9.1[33366]::S0001_pR6K_Amp_2xTY1ce_EGFP_FRT_rpsl_neo_FRT_3xFlag)dFRT::unc-119-Nat) fosmid construct derived from the copy-number inducible vector pCC1Fos grown

up in *E. coli* strain EPI300. Fosmid DNA was extracted and added to a 20 ng/µL DNA mixture containing both the purified fosmid and pCFJ90 P*myo-2*::mcherry. The solution was microinjected into 10-30 adult WT gonads to generate a line of extrachromosomal transgenic arrays. Stable lines were then generated by irradiating with a γ dose of 40 GRAY to produce two lines of the same genotype, PH117, and PH116. The previously produced, PHX893 (F13E9.1(*syb767*) IV) was then crossed 4 times with PX116 to generate the two rescue lines: PHX945 (=DB03) and PHX946 (=DB04) *f13e9.1 (syb767)* IV; *sybIs62* [*f13e9.1*::EGFP::3xFLAG, *unc-119*(+) + P*myo-2*::mCherry].

Generation of UV-Killed OP50 E. coli

32 mL of unconcentrated live OP50 *E. coli* cultures were aliquoted to each 50 mL falcon tube, mixed with absolute ethanol (8 mL) to yield a 20% ethanol solution as described in Calvert et al., 2016 and pipetted into T160 flasks to a volume of 150 ml. Flasks were transferred to a UV-Linker machine (CL-1000 Ultraviolet crosslinker UVP) and irradiated for 120 minutes at 999,900 microjoules/cm^2. 50 ml aliquots of UV-killed *E. coli*-ethanol solution *were* pelleted at 3000 rpm for 10 minutes and resuspended to a 10X concentrate in 5 mL Lennox LB broth.

Drug Treatment

All drug treatments, unless otherwise stated, were administered to *C. elegans* via the addition of the respective compound to NGM (Zheng et al. 2013). Aliquots of stock solutions of the drugs (rilmenidine and rapamycin) were prepared by dissolving them into DMSO solvent, such that the final volume in NGM agar did not exceed 1%. NGM agar was cooled to below 65°C after same-day autoclaving, and respective drugs were concomitantly added. Plates were gently swirled to homogenise the solution and left to dry for 1 hour in a hood. 60 µl of 10X concentrated UV-killed OP50 *E. coli* was then spotted to the centre of the plate and again the

plates were left to dry for 30 minutes in a hood. Unless otherwise stated, plates were prepared one day before use.

Manual Lifespan Assay

Well-fed gravid hermaphrodites were L1 synchronised overnight at 20°C, after hypochlorite treatment. The next day, L1s were transferred to NGM agar plates seeded with 10X OP50 *E. coli* and allowed to develop for 52 hours at 20°C until late L4/adult. At L4/adult molt, animals were transferred to 6-well lifespan assay plates, containing 3 ml of NGM per well with test compounds and 100 µg/ml 5-Fluoro-2'deoxyuridine (FUdR) (Calvert et al. 2016). Plates were maintained at 20°C in the dark, wrapped in parafilm to moderate humidity. Animals were counted every 2 to 3 days, and dead ones (failure to respond to no more than 3 "prods" with a platinum pick) were removed at each inspection. 60 µL of UV-killed OP50 was added three times a week to all plates until day 15 of the assay by which point low-food consumption negated the need for additional *E. coli* (Calvert et al. 2016).

For late administration lifespan assays, on day 12, animals were washed of lifespan assay plates with M9 buffer and carefully pipetted onto new drug plates prepared on day 11, seeded with UV-killed *E. coli*, and contained freshly prepared test compounds and 100 µg/ml FUdR.

For RNAi lifespan analyses, L1 synchronised WT *C. elegans* were grown on live OP50 *E. coli* until the late L4 stage. At the L4 stage, animals were then transferred to NGM plates with 100 µM IPTG, 400 µM FUdR, and 50 µg/ml carbenicillin with either 200 µM rilmenidine or vehicle seeded with live *E. coli* (HT115) expressing an empty vector, *bec-1* dsRNA or *lgg-1* dsRNA. Plates were incubated for 1–2 days at room temperature prior to inducing dsRNA expression. Animals were maintained on the same drug plates for the entirety of their lifespan and scored for survival every 2-3 days.

For all lifespan assays, unless otherwise stated, at least 150 animals per genotype and/or condition were used across at least three independent trials. Animals that crawled into the agar or experienced matricidal events were removed from the assay and not included in the life span calculations. The Kaplan-Meir nonparametric method was used for estimating survival curves. For each condition and/or genotype, survival results were pooled. Lifespan statistics were calculated using the OASIS2 online tool (Han et al. 2016). P-values relating to survival differences between populations were calculated using a log-rank (Mantel-Cox method) test.

Automated Lifespan Measurements

Automated survival analysis was performed as described in (Statzer et al. 2020) employing the lifespan machine setup developed by Stroustrup and colleagues (Stroustrup et al. 2013). Briefly, *C. elegans* were age-synchronized using bleach lysis and kept in L1 culture (52h, 20°C), placed on live OP50 until L4 stage, and subsequently transferred to plates containing the drug, FUdR (50 µg/ml) and dead OP50 from L4 to day 4. Lastly, the animals were moved to tight-fitting Petri dishes (BD Falcon Petri Dishes, 50 x 9 mm) containing the drug, dead bacteria, and FUdR and imaged until the end of life.

Rilmenidine hemifumarate was dissolved in 1% DMSO and supplemented to the agar just before pouring to yield 200 µM, 300 µM, and 400 µM final concentrations. When using dead bacteria, the agar was additionally supplemented with Nystatin (44 U/ml) and Penicillin-Streptomycin (50 U/mL).

Air-cooled Epson V800 scanners were utilized for all experiments operating at a scanning frequency of one scan per 30 minutes. To limit condensation, the tight-fitting plates were dried without lids in a laminar flow hood for 40 minutes before starting the experiment. Furthermore, temperature probes (Thermoworks, Utah, U.S.) were used to monitor the temperature on the scanner flatbed and maintain 20.0°C. Animals that left the imaging area during the experiment were censored. Automated lifespan results were validated using manual

lifespan measurements as described in (Ewald et al. 2016) by picking L4 from normal culturing plates onto the corresponding assay plates. Lifespans were calculated from the L4 stage (= day 0).

Feeding rates and Food Uptake

Larval stage, L4 *C. elegans* hermaphrodites were transferred on the freshly prepared OP50 seeded NGM plates containing rilmenidine (200uM) and FuDR (50uM). At day3, pharyngeal pumping rate was measured by counting pharynx movement per minute; considering only the animals moving on bacteria and constantly pumping for the measurements (Ewald et. al. 2016). The animals were then transferred on the plates containing RFP fluorescent beads diluted in OP50 (1:100) and let them feed for 30minutes (Venz et al. 2021). After feeding, animals were picked in M9 Buffer and washed thrice, anaesthetized in 50mM tetramisole and mounted on 2% agarose pads for imaging. The uptake of food was measured by the presence of beads in the intestine. The red fluorescence from beads was captured using an upright bright-field fluorescence microscope (Tritech Research, model: BX-51-F) at 10X. Experiment was performed in three biological replicates. Approximately 30 animals per condition were used for the analysis. A two-tailed Welch's *t*-test was used for statistics analysis.

Protein Extraction

Whole worm protein lysates were prepared as in(Hu et al. 2017) . ~500 day 1 adult worms per condition and genotype were transferred to either empty NGM plates to induce starvation (+ve control) or to UV-killed OP50 *E.coli* NGM plates containing requisite drugs or vehicle (1% DMSO). After 24 hours, worms were suspended in M9 buffer and centrifuged at 1000rpm for 2 minutes to yield a wet pellet. Worms were then re-suspended on ice in 50μl of RIPA buffer with added phosphatase inhibitors (Cell Signalling Technologies #9806) and complete EDTA-free protease inhibitor cocktail (Roche # 04693159001) before sonication: 10 sec ON/1 minute

OFF, 14% amplitude 3 times until the vast majority of animals were dissolved. Lysates were then spun down twice at 13,000g/20minutes 4° C and immediately frozen at -20°C for no more than one week before use.

Western Blot Analysis

Total protein lysate concentrations were quantified using a bicinchoninic acid (BCA) assay (BioRAD) according to the manufacturer's protocol.

Protein lysates were heated for 5 minutes at 95°C, cooled on ice for 2 minutes and pelleted for 3 minutes at 13,000g. 25µg of protein was loaded per well into an 18 well 10% TGX™ Precast Gel (BioRad), submerged in running buffer (25 mM Tris, 192 mM glycine, 0.1% SDS, pH 8.3) and separated at 300V for approximately 30 minutes. Gels were transferred via Trans-Blot® Turbo™ Transfer System to nitrocellulose membranes and blocked for 60 minutes in blocking buffer (Licor Odyssey® Blocking Buffer #P/N 927-40100). Blots were incubated overnight with the primary antibody Phospho-ERK (Cell Signalling Technology, Catalogue number: 9101) at 1:1000 in blocking buffer (Licor Odyssey® Blocking Buffer #P/N 927-40100) (Gee et al. 2013) with gentle rocking. The following morning, blots were washed three times in TBST before being incubated for 1 hour with the secondary antibody IRDye® 800CW Goat anti-Rabbit IgG at a concentration of 1:10,00 in blocking buffer w/ 0.1% tween. This process was then repeated for the loading control, α-tubulin, using anti- alpha Tubulin (ab72910) and IRDye® 680RD Goat anti-Mouse IgG. Changes in ERK phosphorylation are often calculated using phospho-ERK:Total ERK ratios (Nykamp et al. 2008; Kao et al. 2004). However, given the frequent use of ERK as a loading control (Huang et al. 2004; Hwang et al. 2005), we followed previous examples in both worms (Villanueva-Chimal et al. 2017; Chen et al. 2008) and other models (Redshaw & Loughna 2012; Zhou et al. 2015) and calculated changes in phospho-ERK via the surrogate loading control α-tubulin. In a subset of experiments, executed by Shanghai Model Organisms (Shanghai, China), lysates from PHX945 and PHX946 strains

were probed with monoclonal FLAG-tag antibodies (Sigma #F1804) to verify for fosmid F13E9.1 rescue.

Bound antibody was detected using Odyssey® CLx Imaging System. For all immunoblotting, three independent trials for each genotype and/or condition was completed and mean densiometric ratios between loading control and pERK or FLAG minus local median background were calculated using Image Studio Lite as per software guidelines (Licor Biosciences 2013) . Results were considered significant if the magnitude of the %change exceeded the CV by at least x1.5.

Body Size Phenotyping

Synchronized day 1 *C. elegans* hermaphrodites cultured on live OP50 *E. coli* NGM plates were measured for body length and width in brightfield at 10X objective on a Zeiss Axio Observer following paralysis in 20 mM tetramisole. Between 10-20 animals per data point were used. The animal's length was measured from the head (most visibly anterior buccal line) to a position where the tail tapered to a 10 ± 0.5 *μm* diameter (Petzold et al. 2011). Body width measurements were taken from the posterior vulval peak to the corresponding outer edge of the intestinal cuticle (Collins 2007). Measurements were made using the segmented lines function on Image J software normalized to the scale bar.

Developmental Measurements

Bleach synchronized animals that had been halted in L1 starvation for 24h were measured for development following exposure to food source (Schindler et al. 2014). ~ 500 L1s per genotype/condition were placed onto NGM plates containing rilmenidine and spotted with live OP50 *E. coli* and allowed to develop at 20°C. After 24 hours, measurement of development was conducted at different time points: 0 hours (24 hours exposure to the food source), 3 hours, 6 hours, and then 24 hours (48 hours exposure to the food source). Per trial and time

point, 10 animals were immobilized and mounted onto glass slides in 20 mM solution of tetramisole hydrochloride and then imaged at a 10X objective for body length and 60X objective for vulval development in brightfield on a Zeiss Axio Observer. Vulval development was scored using visual identification of late larval stage vulval checkpoints detailed by Schindler (2014). Statistical significance of differences in vulval development was determined by Two-way ANOVA.

Motility Assay

NGM agar plates were firmly tapped onto the microscope to stimulate movement. In responding animals, not impeded by OP50, body bends were counted for 30 seconds in 10 animals for each condition at each time point (5, 10, and 15 days post-L4 molt) per trial across three independent trials to a total of 30 animals per genotype and condition. Mean deterioration in motility per genotype and/or condition was compared by Two- way of repeated ANOVA and Tukey post hoc correction whilst individual time-point comparisons were tested by student's *t*-test.

Thermotolerance and Recovery Assay

Well-fed gravid hermaphrodites were bleach synchronized and resultant embryos were allowed to grow for 52 hours at 20°C on NGM plates seeded with live OP50 *E. coli*. At the late-L4 stage, animals were transferred to seeded UV-killed OP50 *E. coli* NGM plates containing 200-400 μM rilmenidine, or vehicle for 24h. After 24h of the respective drug exposure, plates were upshifted to an incubator preset to 37°C to induce heat shock. After 3 hours, plates were moved to 20°C and scored for survival after a ~20h "recovery period (Kumsta et al. 2017). At least 100 animals per condition across three identically designed independent trials were scored as either dead or alive by their ability to respond to no more than 3 "prods" with a

platinum pick. Percentage survival was calculated, and populations were compared by one-way ANOVA of variance with Tukey post hoc correction.

Measurement of Autophagy

Gravid hermaphrodites were bleach synchronized and resultant embryos were allowed to grow for 52 hours at 20°C on NGM plates seeded with live OP50 *E. coli*. At the late-L4 stage, animals were transferred to either empty NGM plates to induce starvation (+ve control) or to UV-killed OP50 *E. coli* NGM plates containing the requisite drug or vehicle (1% DMSO). Animals were then incubated at 20°C for 24 hours to maximize drug absorption and efficacy (Zheng et al. 2013). After 24 hours, approximately 10 day-1 adults per condition were imaged at a 10X objective on a Zeiss Axio Observer using a 150 ms exposure time. In total, at least 25 images across three independent trials were collected and pooled for each condition (Morselli et al. 2010; Eisenberg et al. 2009). Levels of autophagy induction were quantified using Zen software. Three 1 mm^2 boxes were sequentially assigned to the most fluorescent areas of the posterior intestine for each animal and with set histogram parameters (black: 0 gamma:1.0 white: 1000 or 5000 for highly fluorescent animals), the total number of visible puncta was manually counted in each box, and a mean number of puncta calculated per mm^2 of animal intestine. The median number of puncta per mm^2 posterior intestine for all animals in each condition was calculated and compared using a one-way (ANOVA) variance analysis corrected by the post hoc Bonferroni test.

PolyQ Protein Aggregation Assay

Gravid hermaphrodites were bleached, and synchronized L1s were transferred to NGM plates containing rilmenidine or DMSO. After incubation for the indicated periods of time, approximately 30 animals per condition across three trials were imaged under a Zeiss Axio Observer microscope at 10X objective. The number of polyQ40::YFP aggregates in body wall

muscle was counted using ZenBlue software with set histogram parameters (black: 500 gamma:1.0 white: 5000). Approximately 50 animals across three independent trials were randomly selected for each treatment group and scored for the number of aggregates. Groups were compared by two-factor repeated measures ANOVA and Tukey posthoc correction.

RNA-sequencing Data Processing

RNAseq data of gene expression changes induced by rilmenidine in mouse liver after 1 month of oral administration was obtained from (Tyshkovskiy et al. 2019). In addition, we performed RNA sequencing of corresponding kidney samples from 8 control mice and 4 mice subjected to the drug for 1 month. A previous experiment (Tyshkovskiy et al. 2019) supplied the mouse kidney tissues for the current study. The animal study from which the tissues were generated for the current study was approved by the University of Michigan's Institutional Animal Care and Use Committee, as described (Tyshkovskiy et al. 2019). RNA was extracted from tissues with the PureLink RNA Mini Kit as described in the manufacturer's protocol. Libraries were prepared and sequenced with a 100 bp read length option on the Illumina HiSeq 2500. RNA-seq data is available in GEO (liver data in GSE131868; kidney data in GSE206982).

Quality filtering and adapter removal were performed using Trimmomatic (version 0.32). Processed/cleaned reads were then mapped with STAR (version 2.5.2b) and counted via featureCounts (version 1.5). To filter out genes with a low number of reads, we left only genes with at least 6 reads in at least 66.6% of the samples separately for each tissue. Filtered data was then passed to RLE normalization (Anders & Huber 2010). Differentially expressed genes between control and treated mice were identified using edgeR. For each gene, we calculated the p-value of its logFC in response to rilmenidine compared to control independently for every tissue. We then converted them to $\log_{10}$(p-value) corrected by the sign of regulation, calculated as:

$$-log_{10}(pv) \times sgn(lfc),$$

where *pv* and *lfc* are p-value and logFC of a certain gene, respectively, and *sgn* is signum function (is equal to 1, -1 and 0 if the value is positive, negative and zero, respectively). The resulting pre-ranked list of genes was used to identify significant associations with the biomarkers of aging, lifespan extension and specific intracellular functions.

In-silico **Association Analysis**

To identify functions enriched by genes perturbed by rilmenidine, we performed gene set enrichment analysis (GSEA) (Subramanian et al. 2005) on a pre-ranked list of genes, obtained as described earlier. REACTOME, KEGG, and GO BP from Molecular Signature Database (MSigDB) have been used as gene sets for GSEA. Only functions related to the processes identified in *C. elegans* (insulin, FOXO, ERK and mTOR signaling, autophagy and proteolysis) were chosen for the analysis.

To identify associations between rilmenidine's effect in mice and biomarkers of lifespan-extending interventions and aging, we employed a GSEA-based algorithm developed in our previous study (Tyshkovskiy et al. 2019). First, for every trait, we specified significant genes, using the FDR threshold of 0.05. Among the remaining genes, we selected 1000 genes with the highest absolute logFC and divided them into up- and downregulated genes. These lists were considered as gene sets. Signatures of lifespan-extending interventions were taken from (Tyshkovskiy et al. 2019), while signatures of aging in liver and kidney were identified from GSE132040 dataset derived by Tabula Muris Consortium (Schaum et al. 2020). Then we calculated normalized enrichment scores (NES) separately for the up- and downregulated lists of each gene set as described in (Lamb 2006) and defined the final NES as a mean of the two. To calculate the statistical significance of the NES, we performed a permutation test where we randomly selected gene sets of the same size. p-value of association was calculated as the

frequency of observed final NES being bigger by absolute value than random final NES obtained from 5,000 random permutations. The resulting p-values were further adjusted for multiple hypothesis testing using Benjamini-Hochberg (BH) procedure (Benjamini & Hochberg 1995). Association was considered significant if the adjusted p-value was smaller than 0.1. To visualize the results of the association analysis, we converted adjusted p-values into significance scores as:

$$significance\ score = -log_{10}(adj.pv) \times sgn(NES),$$

where adj.pv and NES are BH adjusted p-value and final NES, respectively.

References

Anders S & Huber W (2010) Differential expression analysis for sequence count data. *Genome Biol* 11, R106. Available at: https://genomebiology.biomedcentral.com/articles/10.1186/gb-2010-11-10-r106 [Accessed September 5, 2021].

Benjamini Y & Hochberg Y (1995) Controlling the False Discovery Rate: A Practical and Powerful Approach to Multiple Testing. *Journal of the Royal Statistical Society: Series B (Methodological)* 57, 289–300. Available at: https://onlinelibrary.wiley.com/doi/10.1111/j.2517-6161.1995.tb02031.x [Accessed September 5, 2021].

Calvert S, Tacutu R, Sharifi S, Teixeira R, Ghosh P & de Magalhaes JP (2016) A network pharmacology approach reveals new candidate caloric restriction mimetics in C. *Ageing Cell* 15, 256–266.

Chen F, Mackerell AD, Luo Y & Shapiro P (2008) Using Caenorhabditis elegans as a model organism for evaluating extracellular signal-regulated kinase docking domain inhibitors. *Journal of cell communication and signaling* 2, 81–92.

Collins TJ (2007) ImageJ for microscopy. *BioTechniques* 43, S25–S30. Available at: https://www.future-science.com/doi/10.2144/000112517 [Accessed September 5, 2021].

Eisenberg T, Knauer H, Schauer A, Büttner S, Ruckenstuhl C, Carmona-Gutierrez D, Ring J, Schroeder S, Magnes C, Antonacci L, Fussi H, Deszcz L, Hartl R, Schraml E, Criollo A, Megalou E, Weiskopf D, Laun P, Heeren G, Breitenbach M, Grubeck-Loebenstein B, Herker E, Fahrenkrog B, Fröhlich K-U, Sinner F, Tavernarakis N, Minois N, Kroemer G & Madeo F (2009) Induction of autophagy by spermidine promotes longevity. *Nat Cell Biol* 11, 1305–1314. Available at: http://www.nature.com/articles/ncb1975 [Accessed September 5, 2021].

Ewald CY, Marfil V & Li C (2016) Alzheimer-related protein APL-1 modulates lifespan through heterochronic gene regulation in Caenorhabditis elegans. *Aging Cell* 15, 1051–1062.

Gee F, Fisher K, Klemstein U & Poulin GB (2013) An RNAi-Based Dimorphic Genetic Screen Identified the Double Bromodomain Protein BET-1 as a Sumo-Dependent Attenuator of RAS-Mediated Signalling B. Lehner, ed. *PLoS ONE* 8, e83659. Available at: https://dx.plos.org/10.1371/journal.pone.0083659 [Accessed July 11, 2022].

Han SK, Lee D, Lee H, Kim D, Son HG, Yang J-S, Lee S-JV & Kim S (2016) OASIS 2: online application for survival analysis 2 with features for the analysis of maximal lifespan and healthspan in aging research. *Oncotarget* 7, 56147–56152. Available at: https://www.oncotarget.com/lookup/doi/10.18632/oncotarget.11269 [Accessed September 5, 2021].

Hu Q, D'Amora DR, MacNeil LT, Walhout AJM & Kubiseski TJ (2017) The Oxidative Stress Response in Caenorhabditis elegans Requires the GATA Transcription Factor ELT-3 and SKN-1/Nrf2. *Genetics* 206, 1909–1922.

Huang H, Muddiman DC & Tindall DJ (2004) Androgens negatively regulate forkhead transcription factor FKHR (FOXO1) through a proteolytic mechanism in prostate cancer cells. *The Journal of biological chemistry* 279, 13866–77.

Hwang S-G, Yu S-S, Ryu J-H, Jeon H-B, Yoo Y-J, Eom S-H & Chun J-S (2005) Regulation of beta-catenin signaling and maintenance of chondrocyte differentiation by ubiquitin-independent proteasomal degradation of alpha-catenin. *The Journal of biological chemistry* 280, 12758–65.

Kao G, Tuck S, Baillie D & Sundaram M V (2004) C. elegans SUR-6/PR55 cooperates with LET-92/protein phosphatase 2A and promotes Raf activity independently of inhibitory Akt phosphorylation sites. *Development* 131, 755–765.

Kumsta C, Chang JT, Schmalz J & Hansen M (2017) Hormetic heat stress and HSF-1 induce autophagy to improve survival and proteostasis in C. *Nature Communications* 8. Available at: https://doi.org/10.1038/ncomms14337.

Lamb J (2006) The Connectivity Map: Using Gene-Expression Signatures to Connect Small Molecules, Genes, and Disease. *Science* 313, 1929–1935. Available at: https://www.sciencemag.org/lookup/doi/10.1126/science.1132939 [Accessed September 5, 2021].

Licor Biosciences (2013) *Tutorial Guide Featuring Image Studio Analysis Software Version 3.1 CLx*,

Morselli E, Maiuri MC, Markaki M, Megalou E, Pasparaki A, Palikaras K, Criollo A, Galluzzi L, Malik SA, Vitale I, Michaud M, Madeo F, Tavernarakis N & Kroemer G (2010) Caloric restriction and resveratrol promote longevity through the Sirtuin-1-dependent induction of autophagy. *Cell Death Dis* 1, e10–e10. Available at: http://www.nature.com/articles/cddis20098 [Accessed September 5, 2021].

Nykamp K, Lee M-H & Kimble J (2008) C. elegans La-related protein, LARP-1, localizes to germline P bodies and attenuates Ras-MAPK signaling during oogenesis. *RNA (New York, N.Y.)* 14, 1378–89.

Petzold BC, Park S-J, Ponce P, Roozeboom C, Powell C, Goodman MB & Pruitt BL (2011) Caenorhabditis elegans Body Mechanics Are Regulated by Body Wall Muscle Tone. *Biophysical Journal* 100, 1977–1985. Available at: https://linkinghub.elsevier.com/retrieve/pii/S0006349511002530 [Accessed July 10, 2022].

Redshaw Z & Loughna PT (2012) Oxygen concentration modulates the differentiation of muscle stem cells toward myogenic and adipogenic fates. *Differentiation* 84, 193–202.

Schaum N, Lehallier B, Hahn O, Pálovics R, Hosseinzadeh S, Lee SE, Sit R, Lee DP, Losada PM, Zardeneta ME, Fehlmann T, Webber JT, McGeever A, Calcuttawala K, Zhang H, Berdnik D, Mathur V, Tan W, Zee A, Tan M, The Tabula Muris Consortium, Almanzar N, Antony J, Baghel AS, Bakerman I, Bansal I, Barres BA, Beachy PA, Berdnik D, Bilen B, Brownfield D, Cain C, Chan CKF, Chen MB, Clarke MF, Conley SD, Darmanis S, Demers A, Demir K, de Morree A, Divita T, du Bois H, Ebadi H, Espinoza FH, Fish M, Gan Q, George BM, Gillich A, Gòmez-Sjöberg R, Green F, Genetiano G, Gu X, Gulati GS, Hahn O, Haney MS, Hang Y, Harris L, He M, Hosseinzadeh S, Huang A, Huang KC, Iram T, Isobe T, Ives F, Jones R, Kao KS, Karkanias J, Karnam G, Keller A, Kershner AM, Khoury N, Kim SK, Kiss BM, Kong W, Krasnow MA, Kumar ME, Kuo CS, Y. Lam J, Lee DP, Lee SE, Lehallier B, Leventhal O, Li G, Li Q, Liu L, Lo A, Lu W-J, Lugo-Fagundo MF, Manjunath A, May AP, Maynard A, McGeever A, McKay M, McNerney MW, Merrill B, Metzger RJ, Mignardi M, Min D, Nabhan AN, Neff NF, Ng KM, Nguyen PK, Noh J, Nusse R,

Pálovics R, Patkar R, Peng WC, Penland L, Pisco AO, Pollard K, Puccinelli R, Qi Z, Quake SR, Rando TA, Rulifson EJ, Schaum N, Segal JM, Sikandar SS, Sinha R, Sit RV, Sonnenburg J, Staehli D, Szade K, Tan M, Tan W, Tato C, Tellez K, Dulgeroff LBT, Travaglini KJ, Tropini C, Tsui M, Waldburger L, Wang BM, van Weele LJ, Weinberg K, Weissman IL, Wosczyna MN, Wu SM, Wyss-Coray T, Xiang J, Xue S, Yamauchi KA, Yang AC, Yerra LP, Youngyunpipatkul J, Yu B, Zanini F, Zardeneta ME, Zee A, Zhao C, Zhang F, Zhang H, Zhang MJ, Zhou L, Zou J, Pisco AO, Karkanias J, Neff NF, Keller A, Darmanis S, Quake SR & Wyss-Coray T (2020) Ageing hallmarks exhibit organ-specific temporal signatures. *Nature* 583, 596–602. Available at: http://www.nature.com/articles/s41586-020-2499-y [Accessed September 5, 2021].

Schindler AJ, Baugh LR & Sherwood DR (2014) Identification of Late Larval Stage Developmental Checkpoints in Caenorhabditis elegans Regulated by Insulin/IGF and Steroid Hormone Signaling Pathways K. Ashrafi, ed. *PLoS Genet* 10, e1004426. Available at: https://dx.plos.org/10.1371/journal.pgen.1004426 [Accessed September 5, 2021].

Statzer C, Meng J, Venz R, Bland M, Robida-Stubbs S, Patel K, Petrovic D, Emsley R, Liu P, Morantte I, Haynes C, Mair WB, Longchamp A, Filipovic M, Blackwell TK & Ewald CY (2020) *ATF-4 and hydrogen sulfide signalling mediate longevity from inhibition of translation or mTORC1*, Physiology. Available at: http://biorxiv.org/lookup/doi/10.1101/2020.11.02.364703 [Accessed September 4, 2021].

Stiernagle T (2006) Maintenance of C. elegans. *WormBook*. Available at: http://www.wormbook.org/chapters/www_strainmaintain/strainmaintain.html [Accessed September 4, 2021].

Stroustrup N, Ulmschneider BE, Nash ZM, López-Moyado IF, Apfeld J & Fontana W (2013) The Caenorhabditis elegans Lifespan Machine. *Nat Methods* 10, 665–670. Available at: http://www.nature.com/articles/nmeth.2475 [Accessed September 5, 2021].

Subramanian A, Tamayo P, Mootha VK, Mukherjee S, Ebert BL, Gillette MA, Paulovich A, Pomeroy SL, Golub TR, Lander ES & Mesirov JP (2005) Gene set enrichment analysis: A knowledge-based approach for interpreting genome-wide expression profiles. *Proceedings of the National Academy of Sciences* 102, 15545–15550. Available at: http://www.pnas.org/cgi/doi/10.1073/pnas.0506580102 [Accessed September 5, 2021].

Tyshkovskiy A, Bozaykut P, Borodinova AA, Gerashchenko MV, Ables GP, Garratt M, Khaitovich P, Clish CB, Miller RA & Gladyshev VN (2019) Identification and Application of Gene Expression Signatures Associated with Lifespan Extension. *Cell Metabolism* 30, 573-593.e8. Available at: https://doi.org/10.1016/j.cmet.2019.06.018.

Venz R, Pekec T, Katic I, Ciosk R & Ewald CY (2021) End-of-life targeted degradation of DAF-2 insulin/IGF-1 receptor promotes longevity free from growth-related pathologies. *Elife* 10, e71335.

Villanueva-Chimal E, Salinas LS, Fernández-Cardenas LP, Huelgas-Morales G, Cabrera-Wrooman A & Navarro RE (2017) DPFF-1 transcription factor deficiency causes the aberrant activation of MPK-1 and meiotic defects in the *Caenorhabditis elegans* germline. *genesis* 55, e23072.

Zheng S-Q, Ding A-J, Li G-P, Wu G-S & Luo H-R (2013) Drug absorption efficiency in Caenorhbditis elegans delivered by different methods. *PLoS One* 8, e56877.

Zhou Y, Yamada N, Tanaka T, Hori T, Yokoyama S, Hayakawa Y, Yano S, Fukuoka J, Koizumi K, Saiki I & Sakurai H (2015) Crucial roles of RSK in cell motility by catalysing serine phosphorylation of EphA2. *Nature Communications* 6, 7679.
