# Supplementary material for: Rilmenidine extends lifespan and healthspan in Caenorhabditis elegans via a nischarin I1‐imidazoline receptor
Source: Aging Cell. 2023 Jan 20;22(2):e13774. doi: 10.1111/acel.13774 (PMC9924948; doi:10.1111/acel.13774)
Supplement: Supplementary file 4 [file ACEL-22-e13774-s003.docx]

Gene Target	Primer Name	Sequences
f13e9.1	F13_1	F: 5’-AGGACCACCAGGAGCTACAT-3’<br>R: 5’ TCACTGTCGGCGATCTGAAA- 3’
f13e9.1	Int	F: 5’-CATGCGATTACCGAACGACT-3’<br>R: 5’-CCATTCGTACGCGTACAGTA- 3’
f13e9.1	Ext	F: 5’- GCTTGAGAGCAGTCAGACGT-3’<br>R: 5’- TTACGAAGGGCCATTCGTAC- 3’
f13e9.1	F13_2	F: 5’- GAGCGGCAGAAGTAATCCGT-3’<br>R: 5’- AGTGAACGACAAGACGACGAA- 3’
WRM061C_F02	FOSMID	F: 5’- TCCTATCAGAAAACTGGTCG -3’<br>R: 5’- GTGGTCTCTCTTTTCGTTGG- 3’
